# Effectiveness of Multifaceted Strategies to Increase Influenza Vaccination Uptake: A Cluster Randomized Trial

**DOI:** 10.1001/jamanetworkopen.2024.3098

**Published:** 2024-03-25

**Authors:** Yiluan Hu, Ruijie Yan, Xuejun Yin, Enying Gong, Xin Xin, Aiyu Gao, Xiaoyan Shi, Jing Wang, Hao Xue, Luzhao Feng, Juan Zhang

**Affiliations:** 1 School of Population Medicine and Public Health, Chinese Academy of Medical Sciences & Peking Union Medical College, Beijing, China; 2 The George Institute for Global Health, University of New South Wales, Newtown, New South Wales, Australia; 3 Faculty of Psychology, Beijing Normal University, Beijing, China; 4 Dongcheng Primary and Secondary School Health Care Center, Beijing, China; 5 Department of Infectious Disease, Dongcheng Center for Disease Control and Prevention, Beijing, China; 6 Stanford Center on China’s Economy and Institutions, Stanford University, Stanford, California; 7 Research Unit of Population Health, Faculty of Medicine, University of Oulu, Oulu, Finland

## Abstract

**Importance:**

Influenza vaccination rates remain low among primary school students and vary by school in Beijing, China. Theory-informed, multifaceted strategies are needed to improve influenza vaccination uptake.

**Objective:**

To evaluate the effectiveness of multifaceted strategies in improving influenza vaccination uptake among primary school students.

**Design, Setting, and Participants:**

This cluster randomized trial was conducted from September 2022 to May 2023 across primary schools in Beijing, China. Schools were allocated randomly in a 1:1 ratio to multifaceted strategies or usual practice. Schools were deemed eligible if the vaccination rates in the 2019 to 2020 season fell at or below the district-wide average for primary schools. Eligible participants included students in grades 2 and 3 with no medical contraindications for influenza vaccination.

**Intervention:**

The multifaceted strategies intervention involved system-level planning and coordination (eg, developing an implementation blueprint, building social norms, and enhancing supervision), school-level training and educating school implementers (eg, conducting a 1-hour training and developing educational materials), and individual-level educating and reminding students and parents (eg, conducting educational activities and sending 4 reminders about vaccination).

**Main Outcomes and Measures:**

The primary outcomes were influenza vaccination uptake at school reported by school clinicians as well as overall vaccine uptake either at school or outside of school as reported by parents at 3 months. Generalized linear mixed models were used for analysis.

**Results:**

A total of 20 schools were randomized. One intervention school and 2 control schools did not administer vaccination on school grounds due to COVID-19, resulting in a total of 17 schools (9 intervention and 8 control). There was a total of 1691 students aged 7 to 8 years (890 male [52.6%]; 801 female [47.4%]) including 915 in the intervention group and 776 in the control group. Of all participants, 848 (50.1%) were in grade 2, and 1209 (71.5%) were vaccinated in the 2021 to 2022 season. Participants in the intervention and control groups shared similar characteristics. At follow-up, of the 915 students in the intervention group, 679 (74.5%) received a vaccination at school, and of the 776 students in the control group, 556 (71.7%) received a vaccination at school. The overall vaccination rates were 76.0% (695 of 915 students) for the intervention group and 71.3% (553 of 776 students) for the control group. Compared with the control group, there was significant improvement of vaccination uptake at school (odds ratio, 1.40; 95% CI, 1.06–1.85; *P* = .02) and overall uptake (odds ratio, 1.49; 95% CI, 1.12–1.99; *P* = .01) for the intervention group.

**Conclusions and Relevance:**

In this study, multifaceted strategies showed modest effectiveness in improving influenza vaccination uptake among primary school students, which provides a basis for the implementation of school-located vaccination programs of other vaccines in China, and in other countries with comparable programs.

**Trial registration:**

Chinese Clinical Trial Registry: ChiCTR2200062449

## Key Points

**Question:**

Can multifaceted strategies at the system, school, and individual level increase influenza vaccination uptake among primary school students?

**Findings:**

In this cluster randomized trial involving 1691 students from 17 schools, the multifaceted strategies significantly improved the influenza vaccination uptake of students aged 7 to 8 years at school and overall uptake either at school or outside of school.

**Meaning:**

These findings suggest a modest effect of multifaceted strategies in improving influenza vaccination uptake and provide insights for the optimization of school-located vaccination programs for other vaccines in China, as well as in other countries with similar contexts and comparable programs.

## Teaser

This cluster randomized trial assesses the effectiveness of multifaceted strategies to improve vaccine uptake among primary school students in Beijing, China.

## Introduction

Seasonal influenza causes 1 billion infections annually, among which 3 million to 5 million are severe cases. Between 0.29 million and 0.65 million seasonal influenza–associated respiratory deaths occur annually,^1^ with highest rates being among children.^2^ Influenza is transmitted rapidly in crowded areas like schools,^3,4^ and more than 90% of influenza outbreaks in China occur in schools and childcare facilities.^5^ School-aged children are also an important transmitter of influenza in households and communities.^6,7^ Annual influenza vaccinations are recommended as the most effective way to prevent influenza infection and potentially serious complications.^7,8^

School-located influenza vaccination (SLIV) is a cost-effective approach to expand vaccination coverage^9,10,11,12^ and has been adopted by countries including Canada, the UK, Australia, and the US. Since 2007, the Department of Health and the Department of Education of Beijing, China have collaborated to administer free influenza vaccinations in primary and middle schools, and details have been published elsewhere.^13^ However, influenza vaccination coverage remains low. For example, the rates among primary and middle school students were 46.8% during the 2017 to 2018 season.^14^ Additionally, the vaccination rates vary across schools. For example, the rates among primary schools ranged from 30.6% to 76.7%, with over one-half of schools having less than 50% of students vaccinated during the 2019 to 2020 season. However, herd immunity for unvaccinated students may occur in schools with influenza vaccination coverage approaching 50%.^14,15,16,17^ Consequently, viable implementation strategies are needed.

Participation in SLIV is influenced by individual-, intrapersonal-, organizational-, and system-level factors such as parental influenza vaccine hesitancy,^18,19,20,21,22^ social norms,^23^ SLIV program organization,^24,25,26,27^ and COVID-19.^28,29,30,31^ However, approaches to increase influenza vaccination uptake typically include traditional interventions, such as education and financial incentives, and behavioral interventions, such as setting default options, sending reminders, and creating implementation intentions,^32,33,34,35,36,37,38^ with little focus on system-based approaches to improve the performance of SLIV. Additionally, the existing literature on influencing factors and interventions has been mainly conducted in the context of high-income countries,^24,39^ while evidence in China is limited.

In this cluster randomized trial conducted in Beijing, China, theory-informed, system-based, multifaceted strategies were developed to improve the performance of SLIV and the uptake of influenza vaccination. The present study endeavored to elucidate the transformative potential of the multifaceted, enhanced SLIV (E-SLIV) strategies in improving influenza vaccination uptake vs usual practice.

## Methods

### Study Design and Participants

The study was approved by the Institutional Ethics Committee of the Chinese Academy of Medical Sciences and Peking Union Medical College and followed the Consolidated Standards of Reporting Trials (CONSORT) reporting guideline. This study used a 2-group parallel cluster randomized design; the protocol is available in Supplement 1. Schools were deemed eligible if the vaccination rates in the 2019 to 2020 season fell at or below the district-wide mean for primary schools (<65%). To improve representativeness, classes were eligible if the rates in the previous influenza season fell at the grade-wide mean. Given that the rates in previous season for grade 1 were unknown and that students in higher grades were under higher academic pressure, we recruited classes in grade 2 and 3, where the average age of the students was 7 to 8 years old. All students with parental electronic written informed consent to participate in the study were enrolled in early September 2023 after excluding those with medical contraindications for influenza vaccination.

### Randomization and Blinding

Twenty schools were randomly allocated in a 1:1 ratio to either multifaceted E-SLIV strategies or continued usual practice. The allocation sequence was generated by computer using a simple random sampling method by an independent person (E.G.) who was blinded to schools. Random assignment was concealed from the research team until the baseline survey was completed. Given the nature of the intervention, blinding participants and individuals who administered the intervention was unfeasible, but outcome assessors were blinded to the allocation.

### Interventions and Control

Control schools arranged SLIV as usual (including scheduling school vaccination dates, preparing health education materials, sending standard immunization informed consent forms for vaccination, and setting up temporary SLIV clinics) on their own, with irregular supervision from the Department of Education. Intervention schools delivered multifaceted E-SLIV strategies from September 2022 to November 2022. The E-SLIV strategies were developed based on the Consolidated Framework for Implementation Research (CFIR)—Expert Recommendations for Implementing Change Matching tool^40,41,42^ in response to identified barriers under the CFIR.^13^ The barriers included lack of planning and cosmopolitanism, inadequate access to knowledge and information about the SLIV among school implementers, and misconceptions and unmet needs among parents regarding influenza-related information.^13^ The corresponding E-SLIV implementation strategies included planning and coordination at the system level, training and educating school administrators and clinicians at the school level, and educating and reminding students and parents at the individual level (Table 1). Quality control methods to improve fidelity and retention included top-down supervision; a shared, real-time school doctor checklist; and class headteacher prompts.

**Table 1.** Description of the Multifaceted E-SLIV Strategies and Components

Implementation strategies and components	Descriptions of the content, temporality, and dose	Implementers
**System level: planning and coordination**		
Developing a shared, real-time checklist of SLIV activities as an implementation blueprint	The research team developed a shared, real-time checklist, which listed activities that needed to be completed and key milestones, as an implementation blueprint to assist school clinicians in planning the SLIV program. The checklist activities included (1) setting a goal for influenza vaccination rate, (2) coordinating the school vaccination date, and (3) educating and reminding students and parents. During the intervention period (September 2022 to November 2022), school clinicians were asked to plan in advance and update the checklist when carrying out relevant activities.	Research team and school clinicians
Building a social norm	The checklist was shared with all school clinicians in the intervention group, allowing school clinicians to be aware of the adoption and implementation of intervention activities among other schools, thus forming a social norm.	School clinicians
Enhancing supervision and feedback	The responsible unit of the Department of Education was asked to check the shared, real-time checklist twice a week and provide targeted feedback online or onsite to monitor the progress, especially when school clinicians did not comply with the study manual.	Department of Education and school clinicians
**School level: training and educating school administrators and clinicians**		
Conducting a meeting	A 1-hour meeting was held in early September 2022 which included (1) a brief introduction of the whole program for raising school administrators’ and school clinicians’ awareness of the importance of vaccination and improving SLIV performance, (2) key messages about influenza and influenza vaccination, and (3) a demonstration of how to use the shared, real-time checklist and how to use educational materials to better educate and remind students and parents.	Department of Health and Department of Education
Developing and distributing educational materials	The materials were developed based on the Health Belief Model and 3C model (confidence, complacency, and convenience) proposed by the World Health Organization Strategic Advisory Group of Experts and included (1) 1 electronic notification letter with a 3-minute video and a specially designed question based on nudge theory for educating parents and collecting information on their vaccination willingness; (2) 2 audio recordings for broadcasting to students; (3) 1 set of educational slides containing 2 cartoon videos and an interactive quiz, 1 empty poster, and a set of stickers for conducting health education courses for students and parents; (4) 3 videos produced by experts for educating parents; and (5) 4 messages for reminding parents about vaccination.	Department of Health and Department of Education
**Individual level: engaging students and parents**		
Distributing a notification letter	An electronic notification letter was sent to parents in early September 2022 to assess their willingness to get their children vaccinated. The letter contained an easy to understand, 3-minute video to facilitate the understanding of the complicated, scientific immunization informed consent form for vaccination to help parents make an informed decision. In addition, the letter contained a specially designed question based on the nudge theory that highlighted potential consequences of vaccination or nonvaccination to help parents make their decision. Parents had 2 response options: (1) “ I want to vaccinate my children to help protect them against infection with influenza virus and to reduce the spread of infection to other children and adults,” or (2) “I do not want to vaccinate my children, even if it means there may be a higher risk of infection with influenza virus and spread it to other children and adults.”	School clinicians and class headteachers
Broadcasting on campus	Before sending the influenza vaccination informed consent form for vaccination, school clinicians broadcasted twice on campus using the 2 provided audio recordings.	School clinicians
Conducting a health education course	Before sending the influenza vaccination informed consent form for vaccination, school clinicians and class headteachers conducted a health education course where they (1) used 2 cartoon videos in the provided slides to deliver key information about influenza and influenza vaccines, (2) used the interactive quiz in the provided slides to enhance understanding, (3) distributed stickers and asked students to design a poster with their parents, (4) sent 3 videos to educate parents, and (5) involved parents in a student-parent collaborative homework.	School clinicians and class headteachers
Sending reminders	A total of 4 reminders were sent to parents. The first message was sent to remind parents to return the influenza vaccination informed consent form 3 days in advance. The second message was sent to remind students to be prepared for vaccination at school a day before the school vaccination date. The third and fourth messages were sent for reminding parents of unvaccinated students to get their children vaccinated outside of school (in most cases at community health centers) by themselves if their children missed the school influenza vaccination date. These third and fourth messages were sent a day and a week after the school vaccination date, respectively.	School clinicians and class headteachers

Abbreviation: SLIV, school-located influenza vaccination.

#### System Level: Planning and Coordination

The system-level strategy facilitated the planning and coordination processes. These processes included developing a real-time checklist of SLIV activities as an implementation blueprint to assist systematic planning, sharing the checklist with all school clinicians in the intervention group to form a social norm, and tasking the responsible unit of the Department of Education with checking the checklist twice weekly and providing feedback to school clinicians to monitor the progress.

#### School Level: Training and Educating School Administrators and Clinicians

The school-level strategy was to train and educate school administrators and clinicians. Training including conducting a 1-hour meeting with school administrators and clinicians in early September 2022 and distributing educational materials (eg, a notification letter, audio recordings, videos, stickers, and reminder messages) to school clinicians for educating and reminding students and parents.

#### Individual Level: Educating and Reminding Students and Parents

School clinicians and class headteachers educated students and parents. Education included distributing an electronic notification letter, broadcasting on campus, offering a health education course to students, sending 3 educational videos to parents, involving parents in student-parent collaborative homework, and sending 4 messages on different occasions to remind parents of vaccination.

### Outcomes and Data Collection

The primary outcomes were influenza vaccination uptake at school on the school vaccination date as reported by school clinicians and the overall influenza vaccination uptake at school and vaccination uptake outside of school by November 30, 2022 (the end of free influenza vaccination program service provision), as reported by parents. Secondary outcomes included (1) parental influenza-related knowledge, (2) parental influenza vaccine hesitancy assessed by a validated standardized scale (the Chinese version of the Vaccine Hesitancy Scale for Influenza),^43^ (3) parental intention to get their children vaccinated in the 2023 to 2024 season, (4) having influenza-like symptoms, (5) number of medical visits, (6) number of days of school absenteeism among students, and (7) number of days of work absenteeism due to influenza-like symptoms among parents (eTable 1 in Supplement 2). Adverse events included any harm or unexpected adverse events related to intervention and adverse local reactions or systemic reactions after influenza vaccination, given that the intervention was largely behaviorally based and was generally of low risk.^44^ We used the school doctor checklist to measure whether schools compliably delivered the intervention. The primary outcomes and the first 3 secondary outcomes were assessed at the 3-month follow-up in December 2022 (after the end of the free influenza vaccination program service provision on November 30, 2022), while other secondary outcomes were assessed at the 8-month follow-up in May 2023 (the end of the influenza season).

### Sample Size

We estimated that a sample size of 2100 students in 20 schools (10 schools in the intervention and 10 schools in the control group) would provide the trial with 80% power to detect a proportional 20% difference or more in influenza vaccination uptake between students in intervention schools and those in control schools. We used a 2-sided α level of .05, assuming an intracluster correlation of 0.1 and 20% attrition.

### Statistical Analysis

Data were analyzed based on the modified intention-to-treat principle that included all participants assigned to groups and whose data on primary outcomes were available.^45,46,47,48^ To determine the effect size, the generalized mixed effect models used a logit link and restricted maximum likelihood method with adjustment for school-level random intercepts; influenza vaccination uptake in the 2021 to 2022 season; performance of community health centers; school clinicians’ working years; students’ grade and health status; and parental age, educational attainment, and occupation. Predefined exploratory subgroup analysis was conducted by students’ grade, health status, influenza vaccination uptake in the 2021–2022 season, and parental educational attainment and occupation, using *P* values for interaction between the subgroup and treatment variables to examine heterogeneity. Analyses were conducted using SAS version 9.4 (SAS Institute). A 2-sided *P* < .05 was considered statistically significant.

## Results

Of the 3139 students in the 20 recruited schools, 2322 finished the baseline assessments before randomization (Figure). One intervention school and 2 control schools temporarily closed on November 21, 2022, because of COVID-19, and they were unable to administer vaccinations on school grounds, even though they had implemented the rest of the intervention activities as planned. These 3 schools with missing primary outcome data at the end point were excluded from analysis. In addition, 287 students (17.0%) were lost to follow-up at 3 months and 192 students (11.4%) were lost to follow-up at 8 months. A total of 1691 students aged 7 to 8 years (890 male [52.6%]; 801 female [47.4%]) from 17 schools (915 students from 9 intervention schools and 776 students from 8 control schools) were included in the data analysis.

**Figure.**
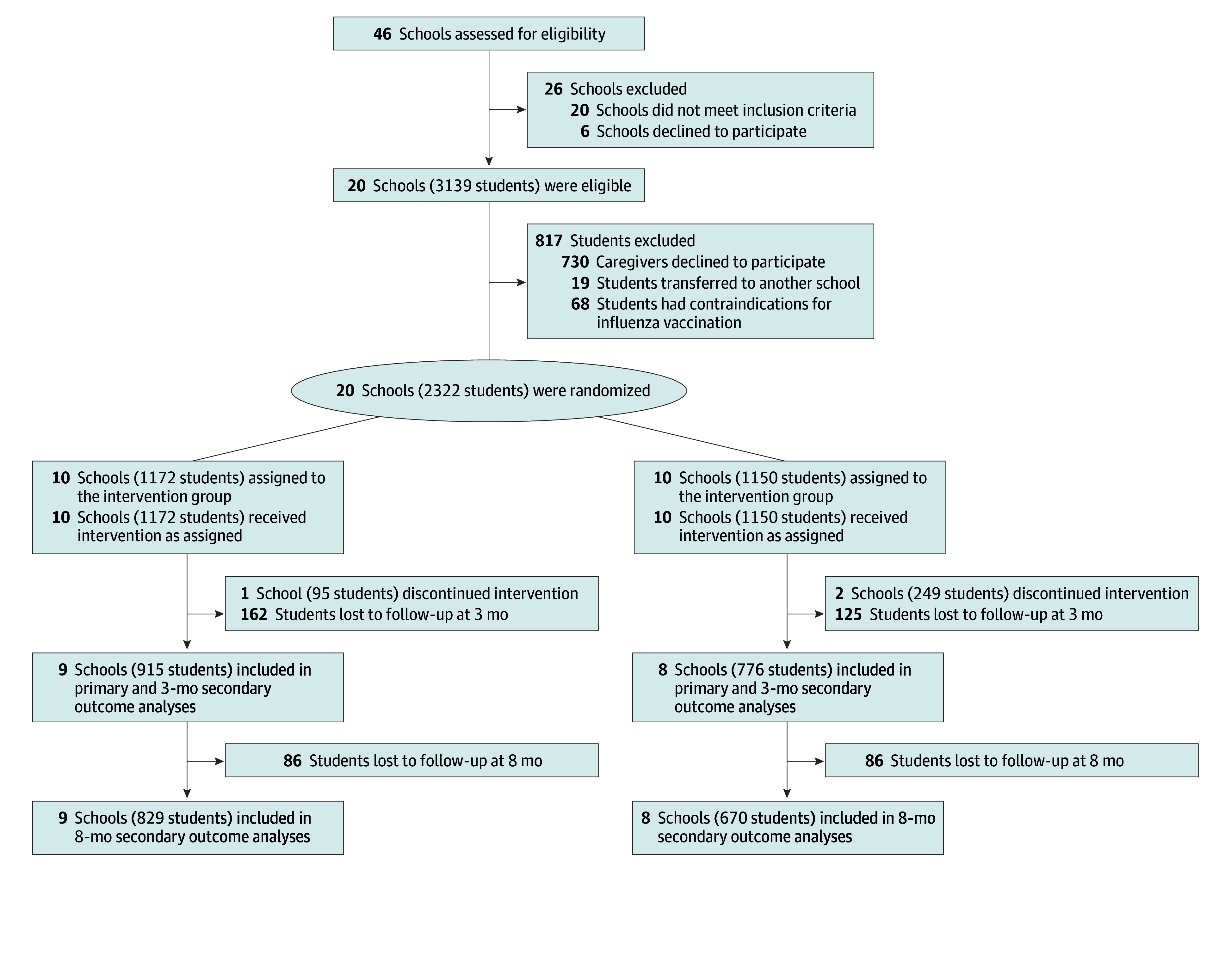
Flow Diagram

### Baseline Characteristics

The baseline characteristics of schools and students in the intervention and control groups were similar (Table 2). Nearly one-half of students (848 students [50.1%]) were in grade 2, 1285 (76.0%) were in good health, and 1209 (71.5%) were administered an influenza vaccine in the 2021 to 2022 season. Of the 1691 parents (mean [SD] age, 39.3 [3.9]) years), 1313 (77.7%) were mothers, 1397 (82.6%) had a bachelor’s degree or above, and 159 (9.4%) were health professionals. The characteristics of the 1691 analyzed students, those from the schools that discontinued intervention (344 students), and those lost to follow-up at 3 months (287 students) were similar (eTable 2 in Supplement 2).

**Table 2.** Baseline Characteristics of Schools and Students

Characteristics	Participants, No. (%)^a^		
	Total (N =1691)	Intervention (n =915)	Control (n =776)
Cluster level			
Schools, No.	17	9	8
No. of students/school, median (range)	108 (58-124)	110 (58-124)	98.5 (66-123)
No. of working-years of the school doctor, median (range)	9 (1-35)	8 (1-35)	11.5 (6-34)
Performance of community health centers, No./Total No.			
Low	1/20 (5.0)	1/10 (10.0)	0
Middle	12/20 (60.0)	6/10 (60.0)	6/10 (60.0)
High	7/20 (35.0)	3/10 (30.0)	4/10 (40.0)
Influenza vaccination rates in the 2019 to 2020 season, median (range), %	47.5 (35.4-61.3)	48.6 (35.4-61.3)	46.6 (42.5-57.3)
Individual level: students			
Grade			
2	848 (50.1)	460 (50.3)	388 (50.0)
3	843 (49.9)	455 (49.7)	388 (50.0)
Sex			
Male	890 (52.6)	478 (52.2)	412 (53.1)
Female	801 (47.4)	437 (47.8)	364 (46.9)
Health status as perceived by parents			
Not good	406 (24.0)	219 (23.9)	187 (24.1)
Good	1285 (76.0)	696 (76.1)	589 (75.9)
Influenza vaccination uptake in the 2021-2022 season			
Yes	1209 (71.5)	678 (70.8)	561 (72.3)
No	482 (28.5)	267 (29.2)	215 (27.7)
Individual level: parents			
Age, mean (SD), y^b^	39.3 (3.88)	39.2 (4.02)	39.4 (3.72)
Family roles			
Father	371 (21.9)	208 (22.7)	163 (21.0)
Mother	1313 (77.7)	701 (76.6)	612 (78.9)
Nonparents	7 (0.4)	6 (0.7)	1 (0.1)
Highest level of educational attainment			
High school diploma	294 (17.4)	165 (18.0)	129 (16.6)
Bachelor’s degree or above	1397 (82.6)	750 (82.0)	647 (83.4)
Occupation			
Health professionals	159 (9.4)	94 (10.3)	65 (8.4)
Non–health professionals	1532 (90.6)	821 (89.7)	711 (91.6)

^a^ Percentages have been rounded and may not total 100.

^b^ Missing values for 2 parents (0.1%) because of parents mistakenly filling in student’s age.

### Primary and Secondary Outcomes

Of the 915 students in the intervention group, 679 (74.5%) received a vaccination at school, and of the 776 students in the control group, 556 (71.7%) received a vaccination at school. Furthermore, when considering vaccination administered either at school or outside of school, the intervention group exhibited a vaccination rate of 76.0% (695 of 915 students), contrasting with the control group rate of 71.3% (553 of 776 students). There was significant improvement of influenza vaccination uptake at school (odds ratio [OR], 1.40; 95% CI, 1.06–1.85; *P* = .02) and overall uptake (OR, 1.49; 95% CI, 1.12–1.99; *P* = .01) (Table 3). Exploratory subgroup analysis revealed that for those who were vaccinated at school, the effect size was greater among those who were vaccinated in the previous influenza season compared with those who were not and those in grade 3 compared with grade 2, although these findings were not significant (Table 4).

**Table 3.** Intervention Effects on Primary and Secondary Outcomes

Outcomes	Intervention, No./Total No. (%)		Control, No/Total No. (%)		Intervention vs control, mean difference (95% CI)^a^
	Baseline	Follow-up	Baseline	Follow-up	
Primary outcomes at 3 months (N = 1691)					
Vaccinated at school^b^	648/915 (70.8)	679/915 (74.5)	561/776 (72.3)	556/776 (71.7)	OR, 1.40 (95% CI, 1.06 to 1.85)^c^
Vaccinated at school or outside of school	648/915 (70.8)	695/915 (76.0)	561/776 (72.3)	553/776 (71.3)	OR, 1.49 (95% CI, 1.12 to 1.99)^c^
Secondary outcomes at 3 months					
Parental influenza-related knowledge, mean (SD)	0.58 (0.24)	0.65 (0.24)	0.59 (0.23)	0.63 (0.24)	0.03 (0.01 to 0.06)^c^
Parental influenza vaccine hesitancy, mean (SD)	2.10 (0.62)	2.01 (0.67)	2.09 (0.59)	2.02 (0.65)	−0.03 (−0.11 to 0.06)
Parental intention to get their children vaccinated in the 2023-2024 season	674/915 (73.7)	701/915 (76.6)	593/776 (76.4)	592/776 (76.3)	OR, 1.30 (95% CI, 0.84 to 2.01)
Secondary outcomes at 8 months (N = 1499)					
Had influenza-like symptoms	NA	187/829 (22.6)	NA	132/670 (19.7)	OR, 1.12 (95% CI, 0.84 to 1.51)
Sought medical visits due to influenza-like symptoms	NA	120/829 (14.5)	NA	84/670 (12.5)	OR, 1.12 (95% CI, 0.79 to 1.59)
No. of medical visits due to influenza-like symptoms, mean (SD)	NA	0.21 (0.67)	NA	0.17 (0.52)	0.03 (−0.03 to 0.10)
No. of days of school absenteeism due to influenza-like symptoms, mean (SD)	NA	0.76 (2.21)	NA	0.74 (1.93)	−0.02 (−0.24 to 0.20)
No. of days of work absenteeism due to influenza-like symptoms, mean (SD)	NA	0.40 (1.46)	NA	0.40 (1.36)	−0.02 (−0.17 to 0.13)

Abbreviations: NA, not applicable; OR, odds ratio.

^a^ Mixed-effect models allowing for the school-clustering effect were used to analyze outcomes, with adjustment for baseline values of the outcomes, performance of community health centers, school clinicians’ working years, students’ grade and health status, and parental age, parental highest level of educational attainment, and parental occupation. Number (percentage) data were calculated as OR (95% CI) and mean (SD) as mean between-group difference (95% CI).

^b^ Baseline value for influenza vaccination uptake in schools in the 2021 to 2022 season was not available because school clinicians did not record it, and it was replaced with parental report of the overall influenza vaccination. There were 3 missing values at follow-up (0.2%) because of nonreporting by school clinicians.

^c^ Denotes statistical significance.

**Table 4.** Subgroup Analysis of Intervention Effects on Primary Outcomes

Subgroup	Influenza vaccination uptake at school			Overall influenza vaccination uptake either at school or outside of school		
	Cases, No.	Adjusted OR (95% CI)	*P *for interaction	Cases, No.	Adjusted OR (95% CI)	*P *for interaction
Student grade						
2	598	1.11 (0.76-1.62)	.14	597	1.19 (0.78-1.82)	.09
3	637	1.91 (1.24-2.93)		651	2.05 (1.33-3.17)	
Student health status as perceived by parents						
Not good	281	1.15 (0.67-1.96)	.48	285	2.00 (1.06-3.76)	.31
Good	954	1.51 (1.09-2.11)		963	1.37 (0.99-1.90)	
Student uptake of influenza vaccination of the 2021 to 2022 influenza season						
Vaccinated	1012	1.84 (1.26-2.67)	.05	1018	2.03 (1.39-2.96)	.01
Unvaccinated	223	1.00 (0.63-1.61)		230	1.01 (0.65-1.56)	
Parental highest level of educational attainment						
High school diploma degree	213	0.76 (0.40-1.46)	.05	221	0.94 (0.48-1.86)	.08
Bachelor’s degree or above	1022	1.58 (1.15-2.16)		1027	1.60 (1.18-2.19)	
Parental occupation						
Health professionals	113	3.30 (1.03-10.58)	.10	116	3.21 (1.01-10.22)	.31
Non–health professionals	1122	1.31 (0.97-1.75)		1132	1.42 (1.06-1.90)	

Abbreviation: OR, odds ratio.

There was an increase in parental knowledge about influenza in the intervention group (mean between-group difference, 0.03 points; 95% CI, 0.01-0.06 points; *P* = .02) (Table 3). However, the intervention had no effect on parental influenza vaccine hesitancy, intention to vaccinate their children in the 2023 to 2024 season, influenza-like symptoms, medical visits, school absenteeism, and work absenteeism.

### Adverse Events

There were no reports of any harm or unintended adverse events of intervention activities. The adverse reactions after influenza vaccination were similar between intervention and control groups. Of the 644 vaccinated students in the intervention group at the 8-month follow-up, 44 (6.8%) reported local reactions and 30 (4.7%) reported systemic reactions. Of the 510 vaccinated students in the control group at the 8-month follow-up, 30 (5.9%) reported local reactions and 22 (4.3%) reported systemic reactions.

### Implementation Fidelity

The intervention was adopted by 9 intervention schools included in data analysis and was implemented with high fidelity (>80%) for most of components, except for sending reminders, for which over one-half of schools (5 schools) adjusted the timing and frequency based on their own vaccination timeline and arrangements of community health centers. The school excluded from analysis also had fidelity as high as the remaining schools in the intervention group.

## Discussion

In this cluster trial, theory-informed, context-specific, multifaceted E-SLIV strategies at the system, school, and individual level showed modest effects in increasing influenza vaccination uptake. The intervention also increased parental influenza-related knowledge but did not reduce influenza vaccine hesitancy, school and work absenteeism, or improve intentions to vaccinate their children against influenza.

Our intervention was novel and encompassed multifaceted implementation strategies. Previous studies^24,25,26,49,50,51^ suggested that participation in SLIV was associated with multiple factors, while intensive, multifaceted interventions were scarce.^52^ The multifaceted E-SLIV strategies based on CFIR involved multiple stakeholders to promote collaborative efforts to tackle issues at different levels. This study supports the importance of taking actions based on various interrelated factors and the crucial role of multifaceted strategies in addressing health challenges.^53^ Notably, multifaceted strategies might be more resource-intensive than individual-level, behavioral intervention. This study did not test the cost-effectiveness of the intervention, but future studies may need to focus more on the cost-effectiveness of resource-intensive interventions.

Although the intervention showed significant effectiveness in improving influenza vaccination uptake, the effect size was modest and was larger among those vaccinated in the previous influenza season. On the one hand, the overall influenza vaccination rate was high at 71.5% in the 2021 to 2022 season, which indicated a potential ceiling effect.^54,55^ In such a scenario, there exists a plausible saturation point, beyond which incremental improvements become progressively challenging to attain. This high baseline rate may be attributed to COVID-19 improving parental awareness of respiratory infections (including influenza), which is supported by other literature.^56,57^ Additionally, hesitant parents may have been less interested in participating in the study, resulting in greater participation of parents who were less hesitant about influenza vaccination and higher vaccination rates. On the other hand, prior research^58,59^ suggests that individuals who have previously embraced influenza vaccination are more inclined to continue doing so in subsequent seasons. However, those who harbor deep-seated vaccine hesitancy may prove resistant to informational nudges,^60,61^ a notion reinforced by our finding that enhanced parental knowledge failed to translate into reduced influenza vaccine hesitancy. It is pertinent to acknowledge the limitations of our individual-level health education activities, primarily disseminated through media channels.^62^ In today’s milieu characterized by electronic sensory overload, the efficacy of such media-centric interventions in mitigating vaccine hesitancy may be curtailed.^63^ This finding underscores the potential inadequacy of the dosage and modality used, suggesting the need for more personalized and tailored interventions that resonate more deeply with vaccine-hesitant individuals. Subgroup analysis by grade indicated that the effect size was greater for students in grade 3 (however, the finding was not statistically significant), which may because grade 3 students were more mature, understood the health education information better, and communicated it with their parents, and that parents may have been less worried about the risk of vaccination for older children. This finding also highlights the importance of developing targeted materials and interventions for different audiences.

Moreover, we did not observe significant differences in influenza-like symptoms, medical visits, or school and work absenteeism. However, we did not confirm influenza cases through laboratory testing, necessitating caution in the interpretation of these outcomes. Therefore, the outcomes of our interventions for influenza-related parameters warrant further scrutiny in future investigations.

Overall, the mechanism by which the interventions increased influenza vaccination uptake may lie in the intervention’s facilitation of the organization and performance of SLIV, such that those who wanted to be vaccinated were vaccinated as successfully as possible; however, the interventions may have a limited impact on those who did not want to be vaccinated. Indeed, strong organization, adequate planning, and good communication are so critical that some studies^25,64,65^ have even recommended a dedicated program coordinator. Meanwhile, as knowledge alone may be insufficient to trigger changes in vaccination intentions and behaviors,^66,67^ future studies should focus more on combining innovative, multichannel, multicomponent, context-specific approaches targeted directly at students and parents to mitigate vaccine hesitancy to further improve vaccination uptake.

A major strength of this study was the use of CFIR to develop the multifaceted E-SLIV strategies systematically, which has been suggested to increase the success of implementation.^68^ Additionally, the CFIR enabled us to systematically understand the contexts and use a common language across study components. Moreover, the multifaceted E-SLIV strategies were designed from a system perspective to involve multilevel strategies, in contrast with previous studies^52,69^ that mainly focused on individual-level, behavioral interventions.

### Limitations

This study had some limitations. First, the outcomes were reported by school clinicians or parents, and the latter in particular might have introduced recall bias. Second, the high baseline vaccination rate (71.5%) may have limited the effect size. Third, 3 of 20 schools were closed (interrupting the delivery of influenza vaccination in schools) and were excluded from the analysis, as were 17.0% of participants lost to follow-up at 3 months. However, these participants shared similar baseline characteristics with those included in the data analysis. The excluded schools were highly compliant with the intervention, and the closing of schools was due to the COVID-19 lockdown. Fourth, the secondary and subgroup analyses were exploratory and not powered. Fifth, although we considered that the mechanism of impact may be to facilitate SLIV organization, this hypothesis requires further exploration because the multifaceted E-SLIV strategies were complex interventions. In fact, we have nested the evaluation of implementation outcomes within this trial, and we expect that the results will help to illustrate this in a separate study.

## Conclusions

In this cluster randomized trial, we found that multifaceted E-SLIV strategies (facilitating planning and coordination, enhancing the capacity of school implementers, and educating and reminding students and parents) improved influenza vaccination uptake, although the effect size was moderate and may have been limited by a potential ceiling effect. These findings proffer a compelling case for the development of multifaceted strategies from a systematic perspective in response to multiple interrelated factors. The results also provide viable insights for developing and optimizing other types of school-located vaccination programs in China, as well as other countries with similar contexts and programs, to encompass multiple components.

## Notes

Supplement 1.
Protocol and Statistical Analysis Plan

Supplement 2. **eTable 1.** Outcomes and Data Collection **eTable 2.** Comparison of Baseline Characteristics of Students Included in the Analysis, Students from the Schools that Discontinued Intervention, and Those Lost to Follow-up at Three Months
eReference

Supplement 3.
Data Sharing Statement

## References

[1] Iuliano AD, Roguski KM, Chang HH, ; Global Seasonal Influenza-associated Mortality Collaborator Network. Estimates of global seasonal influenza-associated respiratory mortality: a modelling study. Lancet. 2018;391(10127):1285-1300. doi:10.1016/S0140-6736(17)33293-2 29248255 PMC5935243

[2] Somes MP, Turner RM, Dwyer LJ, Newall AT. Estimating the annual attack rate of seasonal influenza among unvaccinated individuals: a systematic review and meta-analysis. Vaccine. 2018;36(23):3199-3207. doi:10.1016/j.vaccine.2018.04.063 29716771

[3] Finnie TJ, Copley VR, Hall IM, Leach S. An analysis of influenza outbreaks in institutions and enclosed societies. Epidemiol Infect. 2014;142(1):107-113. doi:10.1017/S0950268813000733 23570654 PMC3857146

[4] Mossong J, Hens N, Jit M, . Social contacts and mixing patterns relevant to the spread of infectious diseases. PLoS Med. 2008;5(3):e74. doi:10.1371/journal.pmed.0050074 18366252 PMC2270306

[5] Chinese Center for Disease Control and Prevention. Technical guidelines for seasonal influenza vaccination in China (2023–2024). Accessed January 23, 2024. https://www.chinacdc.cn/jkzt/crb/bl/lxxgm/jszl_2251/202309/P020230905701009356144.pdf

[6] Neuzil KM, Hohlbein C, Zhu Y. Illness among schoolchildren during influenza season: effect on school absenteeism, parental absenteeism from work, and secondary illness in families. Arch Pediatr Adolesc Med. 2002;156(10):986-991. doi:10.1001/archpedi.156.10.986 12361443

[7] World Health Organization. Influenza (seasonal). October 3, 3023. Accessed January 23, 2024. https://www.who.int/news-room/fact-sheets/detail/influenza-(seasonal)

[8] World Health Organization. Global influenza strategy 2019-2030. 2019. Accessed January 23, 2024. https://apps.who.int/iris/handle/10665/311184

[9] Humiston SG, Schaffer SJ, Szilagyi PG, . Seasonal influenza vaccination at school: a randomized controlled trial. Am J Prev Med. 2014;46(1):1-9. doi:10.1016/j.amepre.2013.08.021 24355665

[10] Kwong JC, Pereira JA, Quach S, ; Public Health Agency of Canada/Canadian Institutes of Health Research Influenza Research Network (PCIRN) Program Delivery and Evaluation Group. Randomized evaluation of live attenuated vs. inactivated influenza vaccines in schools (RELATIVES) cluster randomized trial: pilot results from a household surveillance study to assess direct and indirect protection from influenza vaccination. Vaccine. 2015;33(38):4910-4915. doi:10.1016/j.vaccine.2015.07.044 26232348

[11] Szilagyi PG, Schaffer S, Rand CM, . Impact of elementary school-located influenza vaccinations: a stepped wedge trial across a community. Vaccine. 2018;36(20):2861-2869. doi:10.1016/j.vaccine.2018.03.047 29678459

[12] Szilagyi PG, Schaffer S, Rand CM, . School-located influenza vaccinations for adolescents: a randomized controlled trial. J Adolesc Health. 2018;62(2):157-163. doi:10.1016/j.jadohealth.2017.09.021 29248390

[13] Yan R, Yin X, Hu Y, . Identifying implementation strategies to address barriers of implementing a school-located influenza vaccination program in Beijing. Implement Sci Commun. 2023;4(1):123. doi:10.1186/s43058-023-00501-8 37821918 PMC10566160

[14] Zhang L, Yang P, Duan W, Wang Q. Effects of influenza vaccination among primary and secondary schools in Beijing on influenza outbreaks during 2017-2018 influenza season. Int J Virol. 2020;27(1):11-14.

[15] Sun Y, Yang P, Wang Q, . Influenza vaccination and non-pharmaceutical measure effectiveness for preventing influenza outbreaks in schools: a surveillance-based evaluation in Beijing. Vaccines (Basel). 2020;8(4):714. doi:10.3390/vaccines8040714 33271800 PMC7712374

[16] Pannaraj PS, Wang HL, Rivas H, . School-located influenza vaccination decreases laboratory-confirmed influenza and improves school attendance. Clin Infect Dis. 2014;59(3):325-332. doi:10.1093/cid/ciu340 24829215 PMC4155443

[17] Zhang L, van der Hoek W, Krafft T, . Influenza vaccine effectiveness estimates against influenza A(H3N2) and A(H1N1) pdm09 among children during school-based outbreaks in the 2016-2017 season in Beijing, China. Hum Vaccin Immunother. 2020;16(4):816-822. doi:10.1080/21645515.2019.1677438 31596661 PMC7227706

[18] Kang GJ, Culp RK, Abbas KM. Facilitators and barriers of parental attitudes and beliefs toward school-located influenza vaccination in the United States: systematic review. Vaccine. 2017;35(16):1987-1995. doi:10.1016/j.vaccine.2017.03.014 28320592 PMC5401629

[19] Middleman AB, Short MB, Doak JS. Focusing on flu: parent perspectives on school-located immunization programs for influenza vaccine. Hum Vaccin Immunother. 2012;8(10):1395-1400. doi:10.4161/hv.21575 23095868 PMC3660758

[20] Zakhour R, Tamim H, Faytrouni F, Khoury J, Makki M, Charafeddine L. Knowledge, attitude and practice of influenza vaccination among Lebanese parents: a cross-sectional survey from a developing country. PLoS One. 2021;16(10):e0258258. doi:10.1371/journal.pone.0258258 34648535 PMC8516244

[21] Brown DS, Arnold SE, Asay G, . Parent attitudes about school-located influenza vaccination clinics. Vaccine. 2014;32(9):1043-1048. doi:10.1016/j.vaccine.2014.01.003 24440111

[22] Cheung S, Wang HL, Mascola L, El Amin AN, Pannaraj PS. Parental perceptions and predictors of consent for school-located influenza vaccination in urban elementary school children in the United States. Influenza Other Respir Viruses. 2015;9(5):255-262. doi:10.1111/irv.12332 26073870 PMC4548995

[23] Gargano LM, Weiss P, Underwood NL, . School-located vaccination clinics for adolescents: correlates of acceptance among parents. J Community Health. 2015;40(4):660-669. doi:10.1007/s10900-014-9982-z 25528325 PMC11901353

[24] Perman S, Turner S, Ramsay AI, Baim-Lance A, Utley M, Fulop NJ. School-based vaccination programmes: a systematic review of the evidence on organisation and delivery in high income countries. BMC Public Health. 2017;17(1):252. doi:10.1186/s12889-017-4168-0 28288597 PMC5348876

[25] Kassianos G, MacDonald P, Aloysius I, Reynolds A. Implementation of the United Kingdom’s childhood influenza national vaccination programme: a review of clinical impact and lessons learned over six influenza seasons. Vaccine. 2020;38(36):5747-5758. doi:10.1016/j.vaccine.2020.06.065 32703747

[26] Lott J, Johnson J. Promising practices for school-located vaccination clinics–part II: clinic operations and program sustainability. Pediatrics. 2012;129(suppl 2):S81-S87. doi:10.1542/peds.2011-0737G 22383486

[27] Offeddu V, Low MSF, Surendran S, Kembhavi G, Tam CC. Acceptance and feasibility of school-based seasonal influenza vaccination in Singapore: a qualitative study. Vaccine. 2020;38(7):1834-1841. doi:10.1016/j.vaccine.2019.12.020 31862193

[28] Grech V, Borg M. Influenza vaccination in the COVID-19 era. Early Hum Dev. 2020;148:105116. doi:10.1016/j.earlhumdev.2020.105116 32604011 PMC7301816

[29] World Health Organization. WHO and UNICEF warn of a decline in vaccinations during COVID-19. July 15, 2020. Accessed January 23, 2024. https://www.who.int/news/item/15-07-2020-who-and-unicef-warn-of-a-decline-in-vaccinations-during-covid-19

[30] Wang X, Kulkarni D, Dozier M, ; Usher Network for COVID-19 Evidence Reviews (UNCOVER) group. Influenza vaccination strategies for 2020-21 in the context of COVID-19. J Glob Health. 2020;10(2):021102. doi:10.7189/jogh.10.0201102 33312512 PMC7719353

[31] World Health Organization. Guiding principles for immunization activities during the COVID-19 pandemic. March 26, 2020. Accessed January 23, 2024. https://www.who.int/news/item/26-03-2020-guiding-principles-for-immunization-activities-during-the-covid-19-pandemic

[32] Milkman KL, Patel MS, Gandhi L, . A megastudy of text-based nudges encouraging patients to get vaccinated at an upcoming doctor’s appointment. Proc Natl Acad Sci U S A. 2021;118(20):e2101165118. doi:10.1073/pnas.2101165118 33926993 PMC8157982

[33] Sääksvuori L, Betsch C, Nohynek H, Salo H, Sivelä J, Böhm R. Information nudges for influenza vaccination: Evidence from a large-scale cluster-randomized controlled trial in Finland. PLoS Med. 2022;19(2):e1003919. doi:10.1371/journal.pmed.1003919 35139082 PMC8870595

[34] Yokum D, Lauffenburger JC, Ghazinouri R, Choudhry NK. Letters designed with behavioural science increase influenza vaccination in Medicare beneficiaries. Nat Hum Behav. 2018;2(10):743-749. doi:10.1038/s41562-018-0432-2 31406294

[35] Chapman GB, Li M, Colby H, Yoon H. Opting in vs opting out of influenza vaccination. JAMA. 2010;304(1):43-44. doi:10.1001/jama.2010.892 20606147

[36] Patel MS. Nudges for influenza vaccination. Nat Hum Behav. 2018;2(10):720-721. doi:10.1038/s41562-018-0445-x 31406293

[37] Moran WP, Nelson K, Wofford JL, Velez R, Case LD. Increasing influenza immunization among high-risk patients: education or financial incentive? Am J Med. 1996;101(6):612-620. doi:10.1016/S0002-9343(96)00327-0 9003108

[38] Kimura AC, Nguyen CN, Higa JI, Hurwitz EL, Vugia DJ. The effectiveness of vaccine day and educational interventions on influenza vaccine coverage among health care workers at long-term care facilities. Am J Public Health. 2007;97(4):684-690. doi:10.2105/AJPH.2005.082073 17329659 PMC1829357

[39] Grieco L, Melnychuk M, Ramsay A, . Operational analysis of school-based delivery models to vaccinate children against influenza. Health Syst (Basingstoke). 2020;10(3):212-221. doi:10.1080/20476965.2020.1754733 34377444 PMC8330722

[40] Damschroder LJ, Aron DC, Keith RE, Kirsh SR, Alexander JA, Lowery JC. Fostering implementation of health services research findings into practice: a consolidated framework for advancing implementation science. Implement Sci. 2009;4:50. doi:10.1186/1748-5908-4-50 19664226 PMC2736161

[41] Kirk MA, Kelley C, Yankey N, Birken SA, Abadie B, Damschroder L. A systematic review of the use of the Consolidated Framework for Implementation Research. Implement Sci. 2016;11:72. doi:10.1186/s13012-016-0437-z 27189233 PMC4869309

[42] Waltz TJ, Powell BJ, Fernández ME, Abadie B, Damschroder LJ. Choosing implementation strategies to address contextual barriers: diversity in recommendations and future directions. Implement Sci. 2019;14(1):42. doi:10.1186/s13012-019-0892-4 31036028 PMC6489173

[43] Yan R, Xu J, Hu Y, Gong E, Zhang J. Development of a Chinese version of and influenza vaccine hesitancy scale and its validation among parents of elementary school students. Chinese Journal of Vaccines and Immunization. 2022;28(05):569-575.

[44] Horigian VE, Robbins MS, Dominguez R, Ucha J, Rosa CL. Principles for defining adverse events in behavioral intervention research: lessons from a family-focused adolescent drug abuse trial. Clin Trials. 2010;7(1):58-68. doi:10.1177/1740774509356575 20156957 PMC3163837

[45] Abraha I, Montedori A. Modified intention to treat reporting in randomised controlled trials: systematic review. BMJ. 2010;340:c2697. doi:10.1136/bmj.c2697 20547685 PMC2885592

[46] Halterman JS, Fagnano M, Tajon RS, . Effect of the school-based telemedicine enhanced asthma management (SB-TEAM) program on asthma morbidity: a randomized clinical trial. JAMA Pediatr. 2018;172(3):e174938. doi:10.1001/jamapediatrics.2017.4938 29309483 PMC5885835

[47] Rabe KF, Martinez FJ, Ferguson GT, ; ETHOS Investigators. Triple inhaled therapy at two glucocorticoid doses in moderate-to-very-severe COPD. N Engl J Med. 2020;383(1):35-48. doi:10.1056/NEJMoa1916046 32579807

[48] Kang Z, Qin Y, Sun Y, . Multigenetic pharmacogenomics-guided treatment vs treatment as usual among hospitalized men with schizophrenia: a randomized clinical trial. JAMA Netw Open. 2023;6(10):e2335518. doi:10.1001/jamanetworkopen.2023.35518 37801319 PMC10559185

[49] Liao Q, Dong M, Yuan J, . A mixed-methods study to evaluate elementary school staff’s acceptability, delivery challenges, and communication regarding the implementation of school-located influenza vaccination program in Hong Kong. Vaccines (Basel). 2021;9(10):1175. doi:10.3390/vaccines9101175 34696283 PMC8540161

[50] Key Challenges and Opportunities for Implementing School Located Vaccination Clinics for COVID-19 and Influenza. Key challenges and opportunities for implementing school located vaccination clinics for COVID-19 and influenza: roundtables with school nurses and immunization programs. NASN Sch Nurse. 2022;37(1_suppl)(suppl):15S-23S. doi:10.1177/1942602X211064752 34974774

[51] The landscape of state and local school-located vaccination clinics: practices, policies, and lessons learned for providing COVID-19 and routine vaccinations. NASN Sch Nurse. 2022;37(1_suppl)(suppl):3S-14S. doi:10.1177/1942602X211064750 34974775

[52] Szilagyi PG, Casillas A, Duru OK, . Evaluation of behavioral economic strategies to raise influenza vaccination rates across a health system: results from a randomized clinical trial. Prev Med. 2023;170:107474. doi:10.1016/j.ypmed.2023.107474 36870572 PMC11064058

[53] Swanson RC, Cattaneo A, Bradley E, . Rethinking health systems strengthening: key systems thinking tools and strategies for transformational change. Health Policy Plan. 2012;27(Suppl 4)(suppl 4):iv54-iv61. doi:10.1093/heapol/czs090 23014154 PMC3529625

[54] Buchner DM, Larson EB, White RF. Influenza vaccination in community elderly. a controlled trial of postcard reminders. J Am Geriatr Soc. 1987;35(8):755-760. doi:10.1111/j.1532-5415.1987.tb06354.x 3301990

[55] Dionne B, Brett M, Culbreath K, Mercier RC. Potential ceiling effect of healthcare worker influenza vaccination on the incidence of nosocomial influenza infection. Infect Control Hosp Epidemiol. 2016;37(7):840-844. doi:10.1017/ice.2016.77 27098758

[56] Seiler M, Goldman RD, Staubli G, Hoeffe J, Gualco G, Manzano S; Part Of The International Covid-Parental Attitude Study Covipas Group. Parents’ intent to vaccinate against influenza during the COVID-19 pandemic in two regions in Switzerland. Swiss Med Wkly. 2021;151:w20508. doi:10.4414/smw.2021.20508 34002802

[57] Hou Z, Song S, Du F, . The influence of the COVID-19 epidemic on prevention and vaccination behaviors among Chinese children and adolescents: cross-sectional online survey study. JMIR Public Health Surveill. 2021;7(5):e26372. doi:10.2196/26372 33882450 PMC8158530

[58] Boes L, Boedeker B, Schmich P, Wetzstein M, Wichmann O, Remschmidt C. Factors associated with parental acceptance of seasonal influenza vaccination for their children - a telephone survey in the adult population in Germany. Vaccine. 2017;35(30):3789-3796. doi:10.1016/j.vaccine.2017.05.015 28558985

[59] Wu AMS, Lau JTF, Ma YL, Cheng KM, Lau MMC. A longitudinal study using parental cognitions based on the theory of planned behavior to predict childhood influenza vaccination. J Infect Public Health. 2020;13(7):970-979. doi:10.1016/j.jiph.2020.04.009 32418882

[60] Amin AB, Bednarczyk RA, Ray CE, . Association of moral values with vaccine hesitancy. Nat Hum Behav. 2017;1(12):873-880. doi:10.1038/s41562-017-0256-5 31024188

[61] Sadaf A, Richards JL, Glanz J, Salmon DA, Omer SB. A systematic review of interventions for reducing parental vaccine refusal and vaccine hesitancy. Vaccine. 2013;31(40):4293-4304. doi:10.1016/j.vaccine.2013.07.013 23859839

[62] Singh P, Dhalaria P, Kashyap S, . Strategies to overcome vaccine hesitancy: a systematic review. Syst Rev. 2022;11(1):78. doi:10.1186/s13643-022-01941-4 35473819 PMC9044888

[63] Szilagyi PG, Schaffer S, Rand CM, . Text message reminders for child influenza vaccination in the setting of school-located influenza vaccination: a randomized clinical trial. Clin Pediatr (Phila). 2019;58(4):428-436. doi:10.1177/0009922818821878 30600690

[64] Cawley J, Hull HF, Rousculp MD. Strategies for implementing school-located influenza vaccination of children: a systematic literature review. J Sch Health. 2010;80(4):167-175. doi:10.1111/j.1746-1561.2009.00482.x 20433642

[65] Williams V, Rousculp MD, Price M, . Elementary school-located influenza vaccine programs: key stakeholder experiences from initiation to continuation. J Sch Nurs. 2012;28(4):256-267. doi:10.1177/1059840512438776 22427316

[66] Corace K, Garber G. When knowledge is not enough: changing behavior to change vaccination results. Hum Vaccin Immunother. 2014;10(9):2623-2624. doi:10.4161/21645515.2014.970076 25483478 PMC4975060

[67] Li L, Wood CE, Kostkova P. Vaccine hesitancy and behavior change theory-based social media interventions: a systematic review. Transl Behav Med. 2022;12(2):243-272. doi:10.1093/tbm/ibab148 34850217 PMC8848992

[68] Nilsen P. Making sense of implementation theories, models and frameworks. Implement Sci. 2015;10:53. doi:10.1186/s13012-015-0242-0 25895742 PMC4406164

[69] Siddiqui FA, Padhani ZA, Salam RA, . Interventions to improve immunization coverage among children and adolescents: a meta-analysis. Pediatrics. 2022;149(suppl 5):e2021053852D. doi:10.1542/peds.2021-053852D 35503337

