# Supplementary material for: Effectiveness of Multifaceted Strategies to Increase Influenza Vaccination Uptake: A Cluster Randomized Trial
Source: JAMA Netw Open. 2024 Mar 25;7(3):e243098. doi: 10.1001/jamanetworkopen.2024.3098 (PMC10964116; doi:10.1001/jamanetworkopen.2024.3098)
Supplement: Supplement 1. — Protocol and Statistical Analysis Plan [file jamanetwopen-e243098-s001.pdf]

Supplement 1. Protocol and statistical analysis plan

Effectiveness and Implementation of an Enhanced School-Located Influenza Vaccination Program in China: Protocol for a Hybrid Type 2 Cluster Randomized Controlled Trial

Administrative information

Trial registration: Chinese Clinical Trial Registry (ChiCTR), ChiCTR2200062449. Registered on 8 August 2022 – Prospective registered, http://www.chictr.org.cn/showproj.aspx?proj=174409. Registry name: Enhanced School-located Influenza Vaccination Intervention Study

Protocol version: The finalized version before recruitment (version date: Aug 8, 2022).

Funding: China Medical Board (number 20-379).

Principal Investigator: Juan Zhang, School of Population Medicine and Public Health, Chinese Academy of Medical Sciences and Peking Union Medical College, Beijing, China; Email: zhangjuan@sph.pumc.edu.cn

Table of contents

Introduction ... 3
- Background and rationale ... 3
- Objectives ... 3
- Design ... 3

Methods ... 3
- Study setting ... 3
- Eligibility criteria ... 4
  - Eligibility criteria for schools ... 4
  - Eligibility criteria for classes ... 4
  - Eligibility criteria for students and parents ... 5
- Intervention ... 5
  - Intervention development ... 5
  - Intervention components ... 6
  - Control group: usual practice ... 7
  - Quality control ... 7
- Outcomes ... 7
  - Effectiveness outcomes ... 8
  - Implementation outcomes ... 8
- Participant timeline ... 9
- Sample size ... 9
- Recruitment ... 10
- Assignment ... 10
- Statistical analysis ... 10
- Monitoring ... 11

Ethics and dissemination ... 11
- Research ethics approval ... 11
- Consent or assent ... 11
- Confidentiality ... 11
- Declaration of interests ... 11
- Access to data ... 11
- Dissemination policy ... 11

References ... 13

Introduction

Background and rationale

Influenza is an acute respiratory infectious disease caused by the influenza virus. Epidemiologic studies revealed that infection rates are consistently highest among infant and young children [1-3]. In China, over 90% of influenza outbreaks occur in schools and childcare institutions [3]. Students in schools play an essential role in the transmission of influenza to families and broader communities, resulting in hospitalization and death among elderly adults, as well as work absenteeism and productivity losses among their parents [4,5].

Annual influenza vaccination is recommended as the most effective way for preventing infection and potentially reducing clinical severity [5,6], and herd immunity for unvaccinated students may occur in schools with influenza vaccination coverage approaching 50% [7-10]. School-located influenza vaccination (SLIV) is a cost-effective strategy to expand vaccination coverage among students [11-14]. Even though Beijing has implemented free SLIV for primary and secondary students since 2007, influenza vaccination coverage varies greatly among schools and remains as low as 46.8% during the 2017-2018 season [7]. Reliable, high-quality evidenced-based strategies are urgently needed to increase the influenza vaccination coverage and prevent influenza transmission in primary schools in Beijing.

Participation in SLIV among students is influenced by parental influenza vaccine hesitancy [15-19], social norms [20], organization of SLIV program (e.g., planning, scheduling, communication, motivation, etc.) [21-24], and COVID-19 [25-28], etc. However, most studies typically focused on determinants of the SLIV program at the individual level rather than the organization and system levels, especially in low- and middle-income countries [15,21,29]. In addition, approaches to increase influenza vaccination uptake were typically traditional interventions, such as education and financial incentive, and behavioral interventions, such as setting default options, sending reminders, and creating implementation intentions [30-36], with little focus on system-based approaches.

Objectives

We developed theory-informed multifaceted strategies to improve the performance of SLIV and to improve the uptake of influenza vaccination in primary schools in Beijing, China. The objectives were (1) to evaluate the effectiveness of the multifaceted enhanced school-located influenza vaccination (E-SLIV) strategies in improving the uptake of influenza vaccination, and (2) to evaluate the implementation of the multifaceted E-SLIV strategies through quantitative and qualitative methods.

Design

This study in a two-arm parallel group cluster randomized, hybrid type 2 effectiveness-implementation trial [37] to assess the effectiveness and implementation of the multifaceted E-SLIV strategies. The protocol is in accordance with the SPIRIT statement [38].

Methods

Study setting

The study will take place in 20 primary schools in Dongcheng District, the eastern half of the downtown area of Beijing, China [39,40]. The government of Beijing has implemented influenza vaccination program in primary and secondary schools across all the districts or counties of Beijing since 2007. The delivery model in Dongcheng District is displayed in Figure 1. The program delivery calls for a joint effort of the Department of Health and the Department of Education. Under the supervision of the Department of Health, the Center for Disease

Control and Prevention at the Dongcheng District is responsible for (1) carrying out influenza vaccination in collaboration with the Department of Education, (2) coordinating the Community Health Centers to administer influenza vaccination on school grounds, and (3) monitoring and handling possible suspected abnormal reactions [41]. Under the supervision of the Center for Disease Control and Prevention at the Dongcheng District, Community Health Centers collaborate and communicate with schools to set up school-located vaccination clinics, and are responsible for vaccination for students. Dongcheng Primary and Secondary School Health Care Center, affiliated with the Department of Education of the Dongcheng District, is responsible for the organization, communication, and mobilization of influenza vaccination with schools [41]. School doctors are responsible for the whole organization of SLIV in schools, including educating staff and students, guiding class head teachers to inform parents and collect consent form for influenza vaccination.

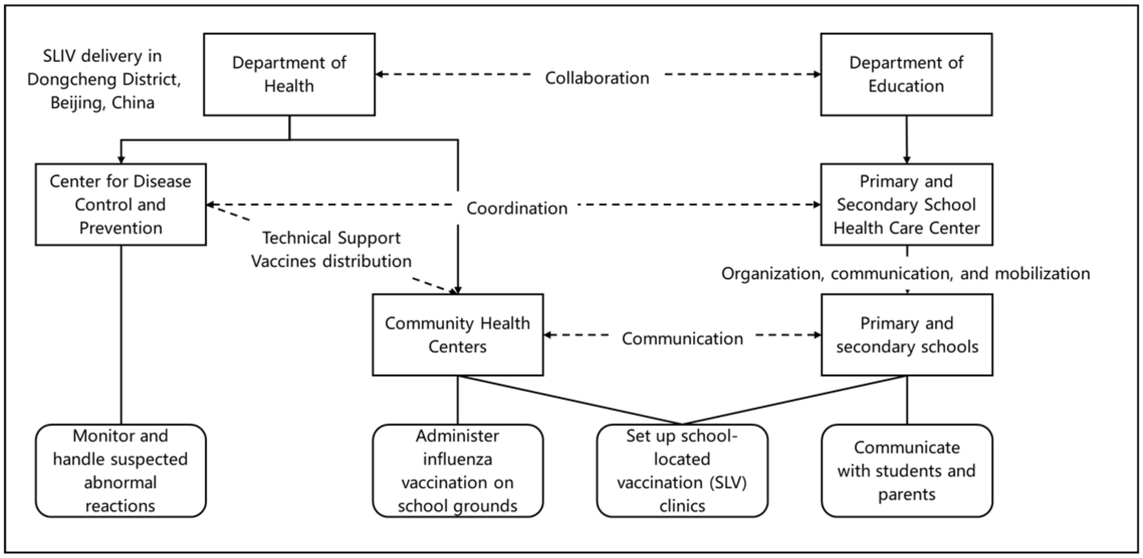

Figure 1 SLIV delivery model in Dongcheng District, Beijing, China

Eligibility criteria

Eligibility criteria for schools

Schools will be included if:

(1) the influenza vaccination rate in the 2019-2020 season was at the average level or below among all primary schools (<65%) in Dongcheng District;

(2) the school administrators and school doctors agree to participate in the study.

Schools will be excluded if:

(1) are boarding schools;

(2) are for minority ethnic groups or children with special skills;

(3) are participating in other similar programs focusing on preventing seasonal influenza;

(4) have plans to merge, split or relocate during the period of implementing this study.

Eligibility criteria for classes

Students in Grade 1 to 6 of primary schools are typically aged 6 to 11 years old, and those aged 5 to 9 years have the highest prevalence of influenza [3]. Given that students in Grade 1 will register after the recruitment of our study, and students at higher grades are under higher academic pressure, our study will only recruit students in Grade 2 and Grade 3 (7 to 8 years old).

Classes will be included if:
(1) are classes in Grade 2 or Grade 3 in the 2022 fall semester;
(2) the influenza vaccination rates in the previous influenza season are at an average level among all classes in the same grade;
(3) the class head teachers agree to participate.

Eligibility criteria for students and parents

Students and parents will be included if:
(1) students without medical contraindications for influenza vaccination;
(2) parents could use smartphone and are in the WeChat (Chinese social software, similar to WhatsApp and Snapchat) group of the class;
(3) parents provide electronic informed consent.
Students and parents will be excluded if students had medical contraindications for influenza vaccination.

Intervention

Intervention development

The multifaceted E-SLIV strategies were developed based on findings in the pilot study and context analysis under the Consolidated Framework for Implementation Research (CFIR), which has been widely used to systematically identify potential barriers and facilitators across diverse scenarios and can be used to design implementation strategies [42,43]. During the 2021-2022 influenza season, we pilot-tested consumer-level strategies developed based on nudge theory [34], which including peer leader, health education, and reminders. The results revealed the limitations of consumer-level strategies, the urgent need for system-based strategies, varied competence of school doctors, and a high level of trust perceived by parents towards schools, education departments, and health departments that deliver the SLIV program. Guided by the CFIR, the context analysis identified the lack of planning and cosmopolitanism, inadequate access to knowledge and information about the SLIV among school implementers, and misconception and unmet needs for influenza-related information among parents as barriers. After inputting these identified barriers, the CFIR - Expert Recommendations for Implementing Change (ERIC) Matching tool [44] outputted a series of ERIC taxonomy: *developing an implementation blueprint*, *promoting network weaving*, *conducting educational meetings*, *developing educational materials*, *distributing educational materials*, *involving parents and family members*, *obtaining and using parents and family feedback*, and *conducting local needs assessment*. We then grouped the single strategies and tailored them to the contexts to finalize the E-SLIV strategies, which included planning and coordination at the system level, training and educating school administrators and doctors at the school level, and educating and reminding students and parents at the consumer level. Figure 2 demonstrates the development of E-SLIV strategies and also the evaluation framework, which will be explicated in the *Outcomes* section.

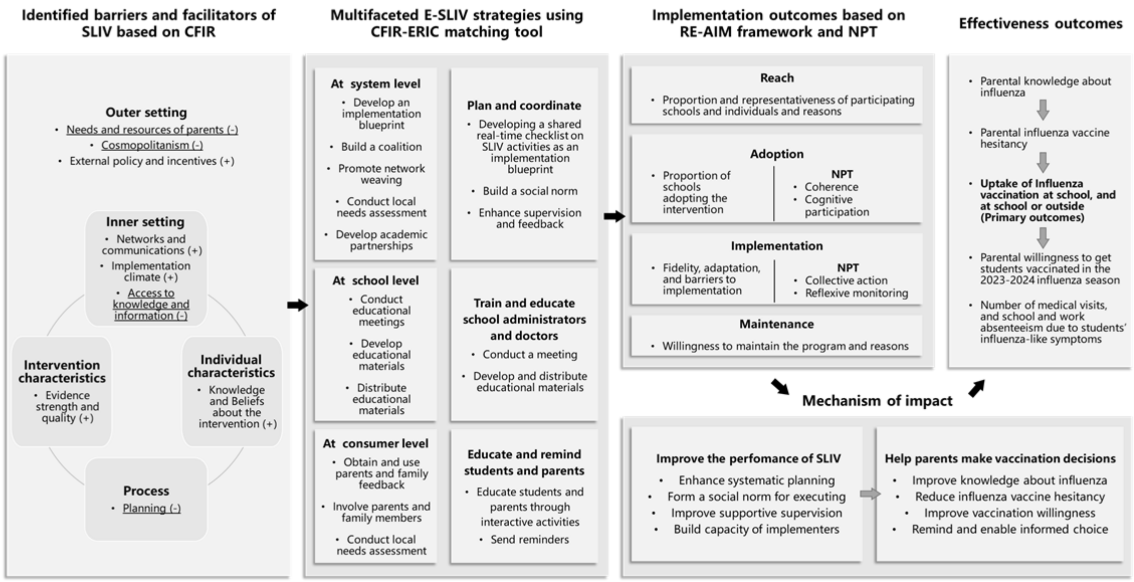

Figure 2. Implementation strategies and evaluation framework

Intervention components

Planning and coordination at the system level:

(1) The research team will build a real-time checklist as an implementation blueprint to assist school doctors in planning SLIV. The checklist lists activities that need to be done, including:

- setting a goal on influenza vaccination rate;
- coordinating school vaccination date;
- educating and reminding students and parents.

(2) School doctors will update the checklist, which would be shared with other school doctors and allow them to be aware of the adoption and implementation of intervention activities, to form a social norm.

(3) The education department will check the checklist twice a week and providing feedback to school doctors to enhance supervision.

Training and educating school administrators and doctors at the school level:

(1) The health department and education department will conduct a one-hour meeting in early September 2022 to school administrators and doctors, and the agenda will include:

- a brief introduction of the whole program for raising awareness to improve the performance of SLIV;
- key messages delivering about influenza and influenza vaccination;
- demonstration of how to use the shared real-time checklist and how to use educational materials to better educate and remind students and parents.

(2) After the educational meeting, the education department will distribute educational materials to help school doctors to educate and remind students and parents. The materials designed for the study were developed based on the Health Belief Model (HBM) [45,46] and "3C" model (Confidence, Complacency, and Convenience) proposed by the World Health Organization Strategic Advisory Group of Experts (WHO SAGE) working group [47,48]. Key messages cover what parents care about most on influenza and flu vaccine, i.e., susceptibility and severity of influenza, impact of influenza on absenteeism, effectiveness and safety of vaccines, convenience of getting vaccinated in schools, and the maturity of the system that delivers vaccines. These educational materials

will include:

- one electronic notification letter with a three-minute video and a specially designed question based on nudge theory [30-32,34] for educating parents and collecting their vaccination willingness;
- two audios for broadcasting to students;
- one educational slides containing two cartoon videos and an interactive quiz, one empty poster, and a set of stickers for conducting health education course to students and parents;
- three videos produced by experts for educating parents;
- four reminders messages for reminding parents.

Educating and reminding students and parents at the consumer level:

(1) The education activities will be conducted before sending the influenza vaccination informed consent form, including:

- distributing the notification letter in early September 2022 to parents;
- broadcasting on campus;
- conducting a health education course to students;
- sending three videos to parents;
- involving parents for student-parent collaborative homework.

(2) Four different reminding messages will be sent at different time points:

- the first will be sent three days before returning the influenza vaccination informed consent form;
- the second will be sent for reminding getting students prepared a day before the school vaccination date;
- the third will be sent for reminding parents of unvaccinated students to get their children vaccinated earlier in Community Health Centers a day after the school vaccination date.
- the fourth, with the same content as the third, will be sent for a week after the school vaccination date.

Control group: usual practice

Ten schools in the control group will continue their usual SLIV practices, which includes preparing materials for publicity and education by school doctors themselves, sending standard immunization informed consent form to parents, and setting up temporary SLV clinics. For ethics consideration, these control schools will receive the same educational materials developed for intervention schools when completing the trial in May 2023 (the end of the 2022-2023 influenza season).

Quality control

A multidisciplinary team of experts in influenza, behavior science, implementation science, design thinking and qualitative methods actively involved in the development of the multifaceted E-SLIV strategies with multi-stakeholders (e.g., parents, school doctors, healthcare providers) to create effective intervention. Four administers from the education department will facilitate the intervention delivery. The top-down supervision of schools by the education department may ensure the delivery of the program and encourage high fidelity. Additionally, the shared real-time school doctor checklist will help the research team and the education department monitor and follow up on the implementation process on time.

Outcomes

This hybrid type 2 effectiveness-implementation study takes a dual focus on effectiveness and implementation of the multifaceted E-SLIV strategies, and the evaluation framework is presented in Figure 2.

Effectiveness outcomes

Primary outcomes

The primary outcomes are (1) the uptake of influenza vaccination at school at the school vaccination date reported by school doctors, and (2) the uptake of influenza vaccination either at school or outside by 30 November 2022 (the end of free influenza vaccination) reported by parents, given that those who fail to get vaccinated in schools may go to Community Health Centers for vaccination.

Secondary outcomes

Secondary outcomes include (1) parents' knowledge about influenza measured by the average score of eight questions, which will be assessed through parent questionnaire at the three-month follow-up (the end of free influenza vaccination); (2) parents' influenza vaccine hesitancy measured by the Chinese Version of the Vaccine Hesitancy Scale for Influenza (VHS-flu-CN), which will be assessed through parent questionnaire at the three-month follow-up; (3) parents' willingness to get their children vaccinated in the 2023-2024 influenza season, which will be assessed through parent questionnaire at the three-month follow-up; (4) students' number of medical visits due to their influenza-like symptoms, which will be assessed through parent questionnaire at the eight-month follow-up (the end of the influenza season); (5) students' number of days of school absenteeism due to their influenza-like symptoms, which will be assessed through parent questionnaire at the eight-month follow-up; and (6) parents' number of days of work absenteeism due to students' influenza-like symptoms, which will be assessed through parent questionnaire at the eight-month follow-up.

Implementation outcomes

Implementation outcomes will be assessed guided by the Reach, Effectiveness, Adoption, Implementation, and Maintenance (RE-AIM) [49,50] framework and the Normalization Process Theory (NPT) [51,52], with the aim to focus both on effectiveness and implementation across multiple essential dimensions and to highlight the importance of incorporating multifaceted E-SLIV strategies into routine SLIV practice. We will integrate the NPT components within the RE-AIM framework, i.e., embed the *coherence* and *cognitive participation* components of the NPT in the *adoption* dimension of the RE-AIM framework, and embed the *collective action* and *reflexive monitoring* components in the implementation dimension.

Reach will be measured by (1) the proportion and representativeness of schools and individuals involved in the study through baseline survey, observation and three-month follow-up surveys, and (2) reasons why some schools and individuals may not be involved through observation and three-month follow-up interviews. Effectiveness will be measured by (1) primary and secondary outcomes as explained in *Effectiveness outcomes*, and (2) individuals' perceptions of effectiveness of intervention components through three-month follow-up surveys and interviews. Adoption will be measured by the proportion of schools adopting the intervention through observation and the real-time school doctor checklist during the intervention period. Implementation will be measured by (1) the proportion of "perfect" intervention delivery completed through observation and the real-time school doctor checklist, and (2) adaptations made to intervention and barriers to implementation through observation, the real-time school doctor checklist, and three-month follow-up interviews. Both adoption and implementation dimensions are from the provider perspective, and the Normalization MeAsure Development questionnaire (NoMAD) [53] will be further utilized to measure providers' perceptions of integrating multifaceted strategies into their routine work. Maintenance will be measured by individual's willingness to maintain the intervention components and reasons.

Participant timeline

The study timeline will span eight months from September 2022 to May 2023, which can be found in Figure 3.

	STUDY PERIOD			
	Enrolment	Allocation	Post-allocation	Close-out
**TIMEPOINT**	***-1 mo to 0***	**0**	***3 mo***	***8 mo***
**ENROLMENT:**				
**Eligibility screen**	X			
**Informed consent**	X			
**Allocation**		X		
**INTERVENTIONS:**				
Intervention group: E-SLIV strategies		◆———	———◆	
Control group: Usual practice		◆———	———◆	
**ASSESSMENTS:**				
Baseline assessment: demographic variables, uptake of influenza vaccination in the 2021-2022 influenza season, influenza-related knowledge, influenza vaccine hesitancy, willingness to get children vaccinated in the 2022-2023 influenza season	X			
Effectiveness outcomes: uptake of influenza vaccination in the 2022-2023 influenza season, influenza-related knowledge, influenza vaccine hesitancy, willingness to get children vaccinated in the 2023-2024 influenza season, students' number of medical visits, number of days of school absenteeism, and number of days of work absenteeism due to students' influenza-like symptoms			X	X
Implementation outcomes: reach, adoption, implementation, and maintenance		◆———	———◆	

Figure 3. Participants timeline of enrolment, interventions, and assessments

Sample size

Our power calculation is based on the comparison of the influenza vaccination rate between intervention group and control group. We estimate that a sample size of 10 schools per arm with 84 students/parents per school

with 0.1 intra-cluster correlation (ICC) would provide 80% statistical power to detect a 20% difference of influenza vaccination rate using a two-sided test at 5% level of significance. Considering the minimal possibility of school-level attrition and a 20% attrition at the student/parent level, we need 10 schools per arm with 105 students/parents per school. The typical size of a class in primary schools in Dongcheng District is about 30 students, so we decided to include 4 classes with 2 in Grade 2 and 2 in Grade 3. Altogether, we aim to recruit a total of 2400 students/parents from 80 classes in 20 schools (10 schools per arm). The sample size calculation was performed by using the Tests for Two Proportions in a Cluster-Randomized Design program in PASS 15.0 software.

Recruitment

First, we contacted the Dongcheng Center for Disease Control and Prevention to get the list of primary schools and the influenza vaccination rates in the 2019-2020 influenza season. Second, we developed the final list of 26 eligible schools out of 45 schools based on the eligibility criteria for schools, and sent invitations to schools in collaboration with Dongcheng Primary and Secondary School Health Care Center. Third, two classes each of Grade 2 and Grade 3 from each school were selected by school doctors according to the eligibility criteria for classes. Altogether, we recruited 20 primary schools and 80 classes. Class head teachers will recruit students and their parents by sending online informed consent forms before administering the baseline survey. Parents who provide electronic informed consent for their children to participate in the study will be enrolled in the study.

Assignment

The randomization occurs at the school level. A total of twenty schools will be randomly allocated 1:1 to either receive multifaceted E-SLIV strategies or continue usual practice. The allocation sequence will be generated by computer using a simple random sampling method. An independent person not involved in the recruitment process will perform random assignment after participants provide informed consent and participate in the baseline survey to achieve allocation concealment and avoid the risk of recruitment bias. Given the nature of the intervention, it may not be possible to blind participants and individuals who administer the intervention.

Statistical analysis

Quantitative and qualitative data will be analyzed independently. For quantitative data, all data collected will be entered into an electronic database with de-identified information. The primary analysis will be based on the intention-to-treat (ITT) principle after checking the data value. We would perform descriptive statistics and inferential statistics, with additional sensitivity analysis and subgroup analysis. Descriptive statistics will be used to present all variables with means and standard deviations or frequencies and percentages as appropriate. Mixed-effect models allowing the adjustment for the school-level clustering effect will be used to compare the effectiveness outcomes of multifaceted E-SLIV strategies between the intervention and control groups. The missing data of variables will be treated with multiple imputations if the percentage of missing data exceeds 5% [54], assuming they are missing at random, and sensitivity analysis will be based on the dataset after multiple imputation. For subgroup analysis, we will examine whether the intervention effect on primary outcomes vary by students' grade, health status, the uptake of influenza vaccination of the 2021-2022 influenza season, and parental highest level of educational attainment and whether they are health professionals, using the interaction terms between each subgroup variable and group assignment variable to assess heterogeneity. As the study is not powered for subgroup analysis, this analysis is considered exploratory. A p value of < 0.05 (two-sided) will be considered statistically significant.

For qualitative data, the interviews will be transcribed and double coded in NVivo 11.0 [55]. Two coders will independently read transcripts to identify preliminary codes. Then, codes with similar meanings will be clustered to form subthemes and themes. The codebook will be constantly checked against the transcripts and finalized by comparison until no new information emerges. All coding results will be compared and discussed between the two coders to reach a consensus. Unsolved discrepancies will be resolved through discussion with senior researchers and at research team meetings. Verbatim quotations of frequently expressed and important themes will be selected and translated from Chinese to English to illustrate the opinions of multi-stakeholders. Quotes will be identified by participants' ID to guarantee anonymity.

Monitoring

Given that intervention period is three months and intervention components are focusing on facilitate behavior change at different levels, there are no anticipated risks from participating. Hence, a data monitoring committee may not be needed. To record any potential adverse events, the number of adverse events after influenza vaccination during the 2022-2023 influenza season will be reported by parents.

Ethics and dissemination

Research ethics approval

This study was approved by the Institutional Ethics Committee for Biomedical Research Projects Involving Human of the Chinese Academy of Medical Sciences & Peking Union Medical College (CAMS&PUMC-IEC-2020-025) on 14 September 2021.

Consent or assent

Electronic informed consent will be obtained from school doctors, class head teachers, and parents.

Confidentiality

All data will be maintained confidential by removing personal identifying information. When reporting the findings, we will aggregate the data and do not use identification of individuals to ensure anonymity.

Declaration of interests

There are no known conflicts of interest.

Access to data

All the data and materials of this study will be available from the corresponding author on reasonable request.

Dissemination policy

The results will be communicated and disseminated through academic publication in peer-reviewed journals, conferences, meetings with stakeholders, etc.

Protocol amendments

An amendment has been made to the protocol on 21 November 2022 when one school in the intervention group and two schools in the control group temporarily closed due to the COVID-19 pandemic, which meant that they were unable to administer influenza vaccination on school grounds and this resulted in missing primary outcome data. These schools were highly compliant to the intervention as assigned and did not intentionally discontinue intervention. Accordingly, we assumed the mechanism causing missing data depended neither on observed data nor on the missing data, and the data were missing completely at random, which may not lead to bias [54,56]. In addition, there is no consensus on how to handle missing data and the most common way to handle missing data in cluster randomized trials is complete case analysis [57]. Hence, we made amendments to the statistical analysis plan: the primary analysis will be based on the modified intention-to-treat principle that include all participants with data on primary outcomes and do not perform imputation for missing outcome data.

References

1. Somes MP, Turner RM, Dwyer LJ, Newall AT. Estimating the annual attack rate of seasonal influenza among unvaccinated individuals: A systematic review and meta-analysis. *Vaccine*. May 31 2018;36(23):3199-3207. doi:10.1016/j.vaccine.2018.04.063

2. Recommendations for Prevention and Control of Influenza in Children, 2021-2022. *Pediatrics*. Oct 2021;148(4)doi:10.1542/peds.2021-053745

3. Chinese Center for Disease Control and Prevention. Technical Guidelines for Seasonal Influenza Vaccination in China(2021–2022). Accessed 6 Aug 2022, https://www.chinacdc.cn/yyrdgz/202109/P020210916330493651111.pdf

4. Neuzil KM, Hohlbein C, Zhu Y. Illness among schoolchildren during influenza season: effect on school absenteeism, parental absenteeism from work, and secondary illness in families. *Arch Pediatr Adolesc Med*. Oct 2002;156(10):986-91. doi:10.1001/archpedi.156.10.986

5. World Health Organization. Seasonal influenza factsheet. Accessed 6 Aug 2022, https://www.who.int/news-room/fact-sheets/detail/influenza-(seasonal)

6. World Health Organization. Global influenza strategy 2019-2030. Accessed Aug 6 2022, https://apps.who.int/iris/handle/10665/311184

7. Zhang L, Yang P, Duan W, Wang Q. Effects of influenza vaccination among primary and secondary schools in Beijing on influenza outbreaks during 2017-2018 influenza season. *International Journal of Virology*. 2020;(01):11-14. doi:10.3760/cma.j.issn.1673-4092.2020.01.003

8. Sun Y, Yang P, Wang Q, et al. Influenza Vaccination and Non-Pharmaceutical Measure Effectiveness for Preventing Influenza Outbreaks in Schools: A Surveillance-Based Evaluation in Beijing. *Vaccines (Basel)*. Dec 1 2020;8(4)doi:10.3390/vaccines8040714

9. Pannaraj PS, Wang HL, Rivas H, et al. School-located influenza vaccination decreases laboratory-confirmed influenza and improves school attendance. *Clin Infect Dis*. Aug 1 2014;59(3):325-32. doi:10.1093/cid/ciu340

10. Zhang L, van der Hoek W, Krafft T, et al. Influenza vaccine effectiveness estimates against influenza A(H3N2) and A(H1N1) pdm09 among children during school-based outbreaks in the 2016-2017 season in Beijing, China. *Hum Vaccin Immunother*. Apr 2 2020;16(4):816-822. doi:10.1080/21645515.2019.1677438

11. Humiston SG, Schaffer SJ, Szilagyi PG, et al. Seasonal influenza vaccination at school: a randomized controlled trial. *Am J Prev Med*. Jan 2014;46(1):1-9. doi:10.1016/j.amepre.2013.08.021

12. Kwong JC, Pereira JA, Quach S, et al. Randomized evaluation of live attenuated vs. inactivated influenza vaccines in schools (RELATIVES) cluster randomized trial: Pilot results from a household surveillance study to assess direct and indirect protection from influenza vaccination. *Vaccine*. Sep 11 2015;33(38):4910-5. doi:10.1016/j.vaccine.2015.07.044

13. Szilagyi PG, Schaffer S, Rand CM, et al. Impact of elementary school-located influenza vaccinations: A stepped wedge trial across a community. *Vaccine*. May 11 2018;36(20):2861-2869. doi:10.1016/j.vaccine.2018.03.047

14. Szilagyi PG, Schaffer S, Rand CM, et al. School-located Influenza Vaccinations for Adolescents: A Randomized Controlled Trial. *J Adolesc Health*. Feb 2018;62(2):157-163. doi:10.1016/j.jadohealth.2017.09.021

15. Kang GJ, Culp RK, Abbas KM. Facilitators and barriers of parental attitudes and beliefs toward school-located influenza vaccination in the United States: Systematic review. *Vaccine*. Apr 11 2017;35(16):1987-1995. doi:10.1016/j.vaccine.2017.03.014

16. Middleman AB, Short MB, Doak JS. Focusing on flu: Parent perspectives on school-located immunization programs for influenza vaccine. *Hum Vaccin Immunother*. Oct 2012;8(10):1395-400. doi:10.4161/hv.21575

17. Zakhour R, Tamim H, Faytrouni F, Khoury J, Makki M, Charafeddine L. Knowledge, attitude and practice of influenza vaccination among Lebanese parents: A cross-sectional survey from a developing country. *PLoS One*. 2021;16(10):e0258258. doi:10.1371/journal.pone.0258258

18. Brown DS, Arnold SE, Asay G, et al. Parent attitudes about school-located influenza vaccination clinics. *Vaccine*. Feb 19 2014;32(9):1043-8. doi:10.1016/j.vaccine.2014.01.003

19. Cheung S, Wang HL, Mascola L, El Amin AN, Pannaraj PS. Parental perceptions and predictors of consent for school-located influenza vaccination in urban elementary school children in the United States. *Influenza Other Respir Viruses*. Sep 2015;9(5):255-62. doi:10.1111/irv.12332

20. Gargano LM, Weiss P, Underwood NL, et al. School-Located Vaccination Clinics for Adolescents: Correlates of Acceptance Among Parents. *J Community Health*. Aug 2015;40(4):660-9. doi:10.1007/s10900-014-9982-z

21. Perman S, Turner S, Ramsay AI, Baim-Lance A, Utley M, Fulop NJ. School-based vaccination programmes: a systematic review of the evidence on organisation and delivery in high income countries. *BMC Public Health*. Mar 14 2017;17(1):252. doi:10.1186/s12889-017-4168-0

22. Kassianos G, MacDonald P, Aloysius I, Reynolds A. Implementation of the United Kingdom's childhood influenza national vaccination programme: A review of clinical impact and lessons learned over six influenza seasons. *Vaccine*. Aug 10 2020;38(36):5747-5758. doi:10.1016/j.vaccine.2020.06.065

23. Lott J, Johnson J. Promising practices for school-located vaccination clinics-- part II: clinic operations and program sustainability. *Pediatrics*. Mar 2012;129 Suppl 2:S81-7. doi:10.1542/peds.2011-0737G

24. Offeddu V, Low MSF, Surendran S, Kembhavi G, Tam CC. Acceptance and feasibility of school-based seasonal influenza vaccination in Singapore: A qualitative study. *Vaccine*. Feb 11 2020;38(7):1834-1841. doi:10.1016/j.vaccine.2019.12.020

25. Grech V, Borg M. Influenza vaccination in the COVID-19 era. *Early Hum Dev*. Sep 2020;148:105116. doi:10.1016/j.earlhumdev.2020.105116

26. World Health Organization. WHO and UNICEF warn of a decline in vaccinations during COVID-19. Accessed Aug 6 2022, https://www.who.int/news/item/15-07-2020-who-and-unicef-warn-of-a-decline-in-vaccinations-during-covid-19

27. Wang X, Kulkarni D, Dozier M, et al. Influenza vaccination strategies for 2020-21 in the context of COVID-19. *J Glob Health*. Dec 2020;10(2):021102. doi:10.7189/jogh.10.021102

28. World Health Organization. Guiding principles for immunization activities during the COVID-19 pandemic. Accessed Aug 6 2022, https://apps.who.int/iris/bitstream/handle/10665/331590/WHO-2019-nCoV-immunization_services-2020.1-eng.pdf?ua=1

29. Cawley J, Hull HF, Rousculp MD. Strategies for implementing school-located influenza vaccination of children: a systematic literature review. *J Sch Health*. Apr 2010;80(4):167-75. doi:10.1111/j.1746-1561.2009.00482.x

30. Milkman KL, Patel MS, Gandhi L, et al. A megastudy of text-based nudges encouraging patients to get vaccinated at an upcoming doctor's appointment. *Proc Natl Acad Sci U S A*. May 18 2021;118(20)doi:10.1073/pnas.2101165118

31. Sääksvuori L, Betsch C, Nohynek H, Salo H, Sivelä J, Böhm R. Information nudges for influenza vaccination: Evidence from a large-scale cluster-randomized controlled trial in Finland. *PLoS Med*. Feb

2022;19(2):e1003919. doi:10.1371/journal.pmed.1003919
32. Yokum D, Lauffenburger JC, Ghazinouri R, Choudhry NK. Letters designed with behavioural science increase influenza vaccination in Medicare beneficiaries. *Nat Hum Behav*. Oct 2018;2(10):743-749. doi:10.1038/s41562-018-0432-2
33. Chapman GB, Li M, Colby H, Yoon H. Opting in vs opting out of influenza vaccination. *Jama*. Jul 7 2010;304(1):43-4. doi:10.1001/jama.2010.892
34. Patel MS. Nudges for influenza vaccination. *Nat Hum Behav*. Oct 2018;2(10):720-721. doi:10.1038/s41562-018-0445-x
35. Moran WP, Nelson K, Wofford JL, Velez R, Case LD. Increasing influenza immunization among high-risk patients: education or financial incentive? *Am J Med*. Dec 1996;101(6):612-20. doi:10.1016/s0002-9343(96)00327-0
36. Kimura AC, Nguyen CN, Higa JI, Hurwitz EL, Vugia DJ. The effectiveness of vaccine day and educational interventions on influenza vaccine coverage among health care workers at long-term care facilities. *Am J Public Health*. Apr 2007;97(4):684-90. doi:10.2105/ajph.2005.082073
37. Curran GM, Bauer M, Mittman B, Pyne JM, Stetler C. Effectiveness-implementation hybrid designs: combining elements of clinical effectiveness and implementation research to enhance public health impact. *Med Care*. Mar 2012;50(3):217-26. doi:10.1097/MLR.0b013e3182408812
38. Chan AW, Tetzlaff JM, Gøtzsche PC, et al. SPIRIT 2013 explanation and elaboration: guidance for protocols of clinical trials. *BMJ*. Jan 8 2013;346:e7586. doi:10.1136/bmj.e7586
39. Hu D, Tang Y, Zheng L, et al. How Parenting and Family Characteristics Predict the Use of Feeding Practices among Parents of Preschoolers: A Cross-Sectional Study in Beijing, China. *Nutrients*. Jul 28 2022;14(15)doi:10.3390/nu14153109
40. Yan R, Gong E, Li X, et al. Impact of Obesogenic Environments on Sugar-Sweetened Beverage Consumption among Preschoolers: Findings from a Cross-Sectional Survey in Beijing. *Nutrients*. Jul 12 2022;14(14)doi:10.3390/nu14142860
41. Beijing Municipal Health Commission. Work plan for influenza vaccination in Beijng in 2021. Accessed 6 Aug 2022, http://wjw.beijing.gov.cn/zwgk_20040/cgxx/202109/t20210914_2492027.html
42. Kirk MA, Kelley C, Yankey N, Birken SA, Abadie B, Damschroder L. A systematic review of the use of the Consolidated Framework for Implementation Research. *Implement Sci*. May 17 2016;11:72. doi:10.1186/s13012-016-0437-z
43. Damschroder LJ, Aron DC, Keith RE, Kirsh SR, Alexander JA, Lowery JC. Fostering implementation of health services research findings into practice: a consolidated framework for advancing implementation science. *Implement Sci*. Aug 7 2009;4:50. doi:10.1186/1748-5908-4-50
44. Waltz TJ, Powell BJ, Fernández ME, Abadie B, Damschroder LJ. Choosing implementation strategies to address contextual barriers: diversity in recommendations and future directions. *Implementation Science*. 2019/04/29 2019;14(1):42. doi:10.1186/s13012-019-0892-4
45. Gargano LM, Herbert NL, Painter JE, et al. Development, theoretical framework, and evaluation of a parent and teacher-delivered intervention on adolescent vaccination. *Health Promot Pract*. Jul 2014;15(4):556-67. doi:10.1177/1524839913518222
46. Flood EM, Rousculp MD, Ryan KJ, et al. Parents' decision-making regarding vaccinating their children against influenza: A web-based survey. *Clin Ther*. Aug 2010;32(8):1448-67. doi:10.1016/j.clinthera.2010.06.020

47. World Health Organization. *Report of the SAGE working group on vaccine hesitancy*. 2014.
48. Schmid P, Rauber D, Betsch C, Lidolt G, Denker ML. Barriers of Influenza Vaccination Intention and Behavior - A Systematic Review of Influenza Vaccine Hesitancy, 2005 - 2016. *PLoS One*. 2017;12(1):e0170550. doi:10.1371/journal.pone.0170550
49. Glasgow RE, Vogt TM, Boles SM. Evaluating the public health impact of health promotion interventions: the RE-AIM framework. *Am J Public Health*. Sep 1999;89(9):1322-7. doi:10.2105/ajph.89.9.1322
50. Glasgow RE, Harden SM, Gaglio B, et al. RE-AIM Planning and Evaluation Framework: Adapting to New Science and Practice With a 20-Year Review. *Front Public Health*. 2019;7:64. doi:10.3389/fpubh.2019.00064
51. May CR, Mair F, Finch T, et al. Development of a theory of implementation and integration: Normalization Process Theory. *Implement Sci*. May 21 2009;4:29. doi:10.1186/1748-5908-4-29
52. Murray E, Treweek S, Pope C, et al. Normalisation process theory: a framework for developing, evaluating and implementing complex interventions. *BMC Med*. Oct 20 2010;8:63. doi:10.1186/1741-7015-8-63
53. Finch TL, Girling M, May CR, et al. Improving the normalization of complex interventions: part 2 - validation of the NoMAD instrument for assessing implementation work based on normalization process theory (NPT). *BMC Med Res Methodol*. Nov 15 2018;18(1):135. doi:10.1186/s12874-018-0591-x
54. Jakobsen JC, Gluud C, Wetterslev J, Winkel P. When and how should multiple imputation be used for handling missing data in randomised clinical trials - a practical guide with flowcharts. *BMC Med Res Methodol*. Dec 6 2017;17(1):162. doi:10.1186/s12874-017-0442-1
55. QSR International Pty Ltd. NVivo qualitative data analysis software version 11.
56. Sterne JA, White IR, Carlin JB, et al. Multiple imputation for missing data in epidemiological and clinical research: potential and pitfalls. *Bmj*. Jun 29 2009;338:b2393. doi:10.1136/bmj.b2393
57. Fiero MH, Huang S, Oren E, Bell ML. Statistical analysis and handling of missing data in cluster randomized trials: a systematic review. *Trials*. Feb 9 2016;17:72. doi:10.1186/s13063-016-1201-z
