# Supplementary material for: Effectiveness of Multifaceted Strategies to Increase Influenza Vaccination Uptake: A Cluster Randomized Trial
Source: JAMA Netw Open. 2024 Mar 25;7(3):e243098. doi: 10.1001/jamanetworkopen.2024.3098 (PMC10964116; doi:10.1001/jamanetworkopen.2024.3098)
Supplement: Supplement 2. — eTable 1. Outcomes and Data Collection eTable 2. Comparison of Baseline Characteristics of Students Included in the Analysis, Students from the Schools that Discontinued Intervention, and Those Lost to Follow-up at Three Months eReference [file jamanetwopen-e243098-s002.pdf]

Supplemental Online Content

Hu Y, Yan R, Yin X, et al. Effectiveness of multifaceted strategies to increase influenza vaccination uptake: a cluster randomized trial. *JAMA Netw Open.* 2024;7(3):e243098. doi:10.1001/jamanetworkopen.2024.3098

eTable 1. Outcomes and Data Collection

eTable 2. Comparison of Baseline Characteristics of Students Included in the Analysis, Students from the Schools that Discontinued Intervention, and Those Lost to Follow-up at Three Months

eReference

This supplemental material has been provided by the authors to give readers additional information about their work.

© 2024 Hu Y et al. *JAMA Network Open.*

1 **eTable 1. Outcomes and Data Collection**

Outcome	Timeline			measurement
	Baseline	3 months	8 months	
**Primary outcomes**				
Influenza vaccination uptake within the school setting on the school vaccination date		√		Reported by school doctors
Overall influenza vaccination uptake within the school setting and outside by 30 November 2022 (the end of free influenza vaccination program service provision)	√[a]	√		Reported by parents
**Secondary outcomes**				
Parental influenza-related knowledge	√	√		Reported by parents, assessed by the average score of eight questions related to influenza (1 point if correct and 0 otherwise)
Parental influenza vaccine hesitancy	√	√		Reported by parents, assessed by the Chinese Version of the Vaccine Hesitancy Scale for Influenza (VHS-flu-CN), in which a high score indicates a higher level of hesitancy [1]
Parental intention to get students vaccinated in the 2023–2024 season	√	√		Reported by parents
Whether students had influenza-like symptoms			√	Reported by parents
Whether students sought medical visits due to influenza-like symptoms			√	Reported by parents
Number of medical visits due to influenza-like symptoms			√	Reported by parents
Number of days of school absenteeism due to influenza-like symptoms			√	Reported by parents
Number of days of work absenteeism due to influenza-like symptoms			√	Reported by parents
**Adverse events**				
Number of adverse reactions after influenza vaccination			√	Reported by parents
Any harm or unexpected adverse events related to intervention activities		√		Reported by school doctors, class headteachers, and parents

2 [a] Influenza vaccination uptake at baseline referred to overall uptake in 2021-2022 season as reported by parents due to a lack of data from school doctors.

© 2024 Hu Y et al. *JAMA Network Open.*

eTable 2. Comparison of Baseline Characteristics of Students Included in the Analysis, Students from the Schools that Discontinued Intervention and those Lost to Follow-up at Three Months

Characteristics [a]	Participants enrolled in randomization					Participants assigned to the intervention group					Participants assigned to the control group				
	Total (n=2322)	Included in analysis (n=1691)	From the schools that discontinued intervention (n=344)	Lost to follow-up at 3 months (n=287)	*P*	Total (n=1172)	Included in analysis (n=915)	From the schools that discontinued intervention (n=95)	Lost to follow-up at 3 months (n=162)	*P*	Total (n=1150)	Included in analysis (n=776)	From the schools that discontinued intervention (n=249)	Lost to follow-up at 3 months (n=125)	*P*
**Students**															
**Grade**					.38					.92					.29
2	1156 (49.8)	848 (50.2)	160 (46.5)	148 (51.6)		593 (50.6)	460 (50.3)	49 (51.6)	84 (51.9)		563 (49.0)	388 (50.0)	111 (44.6)	64 (51.2)	
3	1166 (50.2)	843 (49.9)	184 (53.5)	139 (48.4)		579 (49.4)	455 (49.7)	46 (48.4)	78 (48.2)		587 (51.0)	388 (50.0)	138 (55.4)	61 (48.8)	
**Sex**					.41					.46					.69
Male	1210 (52.1)	890 (52.6)	181 (52.6)	139 (48.4)		601 (51.3)	478 (52.2)	46 (48.4)	77 (47.5)		609 (53.0)	412 (53.1)	135 (54.2)	62 (49.6)	
Female	1112 (47.9)	801 (47.4)	163 (47.4)	148 (51.6)		571 (48.7)	437 (47.8)	49 (51.6)	85 (52.5)		541 (47.0)	364 (46.9)	114 (45.8)	63 (50.4)	
**Health status perceived by parents**					.84					.40					.21
Not good	560 (24.1)	406 (24.0)	81 (23.6)	73 (25.4)		283 (24.1)	219 (23.9)	28 (29.5)	36 (22.2)		277 (24.1)	187 (24.1)	53 (21.3)	37 (29.6)	
Good	1762 (75.9)	1285 (76.0)	263 (76.5)	214 (74.6)		889 (75.9)	696 (76.1)	67 (70.5)	126 (77.8)		873 (75.9)	589 (75.9)	196 (78.7)	88 (70.4)	

© 2024 Hu Y et al. *JAMA Network Open.*

Characteristics [a]	Participants enrolled in randomization					Participants assigned to the intervention group					Participants assigned to the control group				
	Total (n=2322)	Included in analysis (n=1691)	From the schools that discontinued intervention (n=344)	Lost to follow-up at 3 months (n=287)	*P*	Total (n=1172)	Included in analysis (n=915)	From the schools that discontinued intervention (n=95)	Lost to follow-up at 3 months (n=162)	*P*	Total (n=1150)	Included in analysis (n=776)	From the schools that discontinued intervention (n=249)	Lost to follow-up at 3 months (n=125)	*P*
**Parents**															
**Age, Mean (SD), y [b]**	39.3 (3.83)	39.3 (3.88)	39.3 (3.62)	39.2 (3.74)	.93	39.1 (3.99)	39.2 (4.02)	38.1 (4.07)	39.1 (3.67)	.05	39.5 (3.65)	39.4 (3.72)	39.8 (3.33)	39.3 (3.84)	.36
**Family roles [c]**					.10					.36					.24
Father	535 (23.0)	371 (21.9)	86 (25.0)	78 (27.2)		277 (23.6)	208 (22.7)	24 (25.3)	45 (27.8)		258 (22.4)	163 (21.0)	62 (24.9)	33 (26.4)	
Mother	1778 (76.6)	1313 (77.7)	256 (74.4)	209 (72.8)		887 (75.7)	701 (76.6)	69 (72.6)	117 (72.2)		891 (77.5)	612 (78.9)	187 (75.1)	92 (73.6)	
Nonparents	9 (0.4)	7 (0.4)	2 (0.6)	0 (0)		8 (0.7)	6 (0.7)	2 (2.1)	0 (0)		1 (0.1)	1 (0.1)	0 (0)	0 (0)	
**Highest level of educational attainment**					.29					<.001					.006
High school/ Diploma degree	405 (17.4)	294 (17.4)	68 (19.8)	43 (15.0)		236 (20.1)	165 (18.0) [d]	47 (49.5) [e]	24 (14.8) [d]		169 (14.7)	129 (16.6) [d]	21 (8.4) [e]	19 (15.2) [d, e]	
Bachelor’s degree or above	1917 (82.6)	1397 (82.6)	276 (80.2)	244 (85.0)		936 (79.9)	750 (82.0) [d]	48 (50.5) [e]	138 (85.2) [d]		981 (85.3)	647 (83.4) [d]	228 (91.6) [e]	106 (84.8) [d, e]	
**Occupation**					.05					<.001					.60
Health	226 (9.7)	159 (9.4)	28 (8.1)	39 (13.6)		127 (10.8)	94 (10.3) [d]	3 (3.2) [d]	30		99 (8.6)	65 (8.4)	25 (10.0)	9 (7.2)	

© 2024 Hu Y et al. *JAMA Network Open.*

Characteristics [a]	Participants enrolled in randomization					Participants assigned to the intervention group					Participants assigned to the control group				
	**Total (n=2322)**	**Included in analysis (n=1691)**	**From the schools that discontinued intervention (n=344)**	**Lost to follow-up at 3 months (n=287)**	***P***	**Total (n=1172)**	**Included in analysis (n=915)**	**From the schools that discontinued intervention (n=95)**	**Lost to follow-up at 3 months (n=162)**	***P***	**Total (n=1150)**	**Included in analysis (n=776)**	**From the schools that discontinued intervention (n=249)**	**Lost to follow-up at 3 months (n=125)**	***P***
professionals									(18.5) [e]						
Non-health professionals	2096 (90.3)	1532 (90.6)	316 (91.9)	248 (86.4)		1045 (89.2)	821 (89.7) [d]	92 (96.8) [d]	132 (81.5) [e]		1051 (91.4)	711 (91.6)	224 (90.0)	116 (92.8)	

[a] Unless otherwise indicated, data are expressed as number (%). Percentages have been rounded and may not total 100.

[b] Missing values for 2 (0.1%) among students included in primary outcome analysis, 7 (0.3%) among those from the schools that discontinued intervention, and 23 (1.0%) among those lost to follow-up at 3 months because of parents mistakenly filling in student's age.

[c] Excluded "Nonparents" category when performed Pearson χ^2 test

[d, e] Different superscripts indicate a significant difference (*P* < .05) within baseline characteristics (e.g., highest level of educational attainment).

eReference

1. Yan R, Xu J, Hu Y, Gong E, Zhang J. Development of a Chinese version of and influenza vaccine hesitancy scale and its validation among parents of elementary school students. *Chinese Journal of Vaccines and Immunization*. 2022;28(05):569-575. doi:10.19914/j.CJVI.2022109

© 2024 Hu Y et al. *JAMA Network Open.*
