# Supplementary material for: Effectiveness of Multifaceted Strategies to Increase Influenza Vaccination Uptake: A Cluster Randomized Trial
Source: JAMA Netw Open. 2024 Mar 25;7(3):e243098. doi: 10.1001/jamanetworkopen.2024.3098 (PMC10964116; doi:10.1001/jamanetworkopen.2024.3098)
Supplement: Supplement 3. — Data Sharing Statement [file jamanetwopen-e243098-s003.pdf]

Data Sharing Statement

Hu. Effectiveness of Multifaceted Strategies to Increase Influenza Vaccination Uptake. *JAMA Netw Open*. Published March 25, 2024. doi:10.1001/jamanetworkopen.2024.3098

Data
Data available: No

Additional Information
Explanation for why data not available: The data will be available upon reasonable request.
